# Fc‐Dependent Targeting of the GAS6–MERTK Axis With Monoclonal and Bispecific Antibodies Overcomes Drug Resistance Across EGFR Mutations in Exons 19, 20, and 21

**DOI:** 10.1002/advs.78290

**Published:** 2026-10-14

**Authors:** Suvendu Giri, Boobash Raj Selvadurai, Nitin Gupta, Arturo Simoni‐Nieves, Alessandro Genna, Moshit Lindzen, Rishita Chatterjee, Marieke Van Daele, Deepthi Ramesh Kumar, Mirie Zerbib, Roni Oren, Isabelle Sophie Meijer, Elisabeth Franziska Wippich, Yahel Avraham, Rony Dahan, Yuya Haga, Laura E. Kilpatrick, Simon Platt, Stephen J. Hill, Lipika R. Pal, Eytan Ruppin, Yasuo Tsutsumi, Eliane Hadas Yardeni, Muhammad Abu Ahmad, Olga Radinsky, Donatella Romaniello, Mattia Lauriola, Angel Porgador, Yosef Yarden

**Affiliations:** ^1^ Department of Immunology and Regenerative Biology Weizmann Institute of Science Rehovot Israel; ^2^ Department of Veterinary Resources Weizmann Institute of Science Rehovot Israel; ^3^ Department of Molecular Cell Biology Weizmann Institute of Science Rehovot Israel; ^4^ Department of System Immunology Weizmann Institute of Science Rehovot Israel; ^5^ Graduate School of Pharmaceutical Sciences The University of Osaka Osaka Japan; ^6^ Centre of Membrane Proteins and Receptors (COMPARE) University of Birmingham and University of Nottingham Midlands UK; ^7^ Division of Bimolecular Science and Medicinal Chemistry School of Pharmacy Biodiscovery Institute University of Nottingham Nottingham UK; ^8^ Division of Physiology Pharmacology and Neuroscience School of Life Sciences University of Nottingham Nottingham UK; ^9^ Cancer Data Science Laboratory Center for Cancer Research National Cancer Institute Bethesda Maryland USA; ^10^ Graduate School of Medicine The University of Osaka Osaka Japan; ^11^ Institute for Open and Transdisciplinary Research Initiatives The University of Osaka Osaka Japan; ^12^ Global Center for Medical Engineering and Informatics The University of Osaka Osaka Japan; ^13^ R3 Institute for Newly‐Emerging Science Design The University of Osaka Osaka Japan; ^14^ Department of Life Science Core Facility Weizmann Institute of Science Rehovot Israel; ^15^ The Shraga Segal Department of Microbiology Immunology and Genetics Faculty of Health Sciences Ben‐Gurion University of the Negev Beer‐Sheva Israel; ^16^ Department of Medical and Surgical Sciences (DIMEC) University of Bologna Bologna Italy; ^17^ IRCCS Azienda Ospedaliero‐Universitaria di Bologna University of Bologna Bologna Italy

**Keywords:** bispecific antibody, drug resistance, EGFR, GAS6, MERTK, osimertinib

## Abstract

Resistance of lung cancer to EGFR‐targeting tyrosine kinase inhibitors (TKIs) like osimertinib frequently results from secondary mutations or activation of bypass signaling pathways. Re‐analysis of clinical data unveiled elevated expression of GAS6 and its receptor MERTK in lung cancer patients developing resistance to osimertinib. This implied that the GAS6–MERTK axis could represent a therapeutic vulnerability. Functionally, GAS6 overexpression enhanced cancer cell survival, whereas a new anti‐GAS6 antibody delayed tumor relapses in xenograft models. Similarly, ablation of MERTK sensitized lung cancer cells to osimertinib, revealing a bypass crosstalk between EGFR and MERTK, likely taking place within caveolae. An anti‐MERTK antibody was generated, and a bispecific antibody, Bis‐EM, targeting both EGFR and MERTK, was designed. Bis‐EM promoted the degradation of both receptors and displayed the capacity to promote immune‐mediated cell killing. In combination with TKIs, concurrent antibody‐mediated inhibition of MERTK and EGFR suppressed the growth of TKI‐resistant spheroids. Co‐treatment with Bis‐EM and TKIs delayed tumor relapses in an Fc‐dependent manner across patient‐derived xenografts harboring clinically challenging EGFR exon 20 insertions and other mutations. Collectively, these findings identify the GAS6–MERTK axis as an important driver of drug resistance and provide a strong rationale for the clinical development of Bis‐EM.

## Graphical abstract

EGFR mutations drive a substantial fraction of non‐small cell lung cancers (NSCLC) and are effectively targeted by tyrosine kinase inhibitors (TKIs), including osimertinib. However, resistance inevitably develops through secondary mutations and compensatory receptor activation, including the GAS6 receptor MERTK. The newly generated bispecific EGFR×MERTK antibody Bis‐EM suppresses resistance by efficiently promoting receptor degradation and inducing immune‐mediated ADCC.

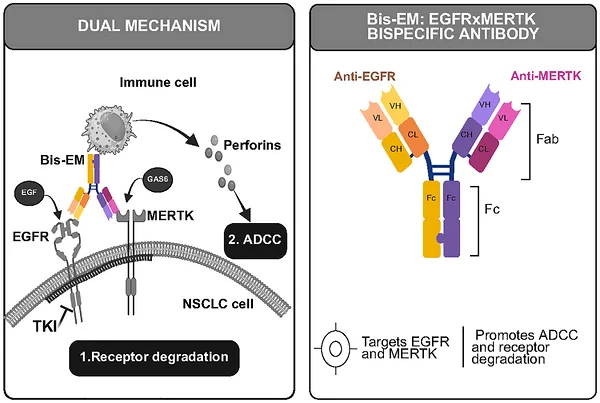

## 1 Introduction

The American Cancer Moonshot initiative, which aimed at improving cancer therapy, recommended that overcoming drug resistance be considered one of the top priorities in oncology [1]. Indeed, the majority of patients with advanced cancer die either because their cancers are inherently resistant to anti‐cancer drugs, or their cancers initially respond to specific drugs but they later develop tolerance [2]. Resistance universally limits the application of not only chemotherapeutic agents but also kinase inhibitors [3], anti‐receptor antibodies, and immune checkpoint inhibitors [4]. Lung cancer provides an example relevant to the importance of understanding and overcoming resistance to anti‐cancer drugs. This malignancy is the leading cause of oncology‐related deaths, and over 75% of all cases are classified as NSCLC (non‐small cell lung cancer). A significant fraction of NSCLC patients present activating mutations in the epidermal growth factor receptor (EGFR) [5, 6, 7, 8, 9]. The most common mutations are exon 19 deletions, an exon 21 point mutation, L858R, and diverse exon 20 aberrations [10].

In clinical trials, the first‐generation EGFR‐specific TKIs achieved superiority in comparison to chemotherapy [11, 12, 13]. However, despite initial responses, patients inevitably become resistant within 10–20 months. The most common mechanism of resistance involves emergence of the T790M secondary mutation, which increases EGFR's affinity toward ATP [14]. Other mechanisms include adaptive up‐regulation of *MET* [15], *HER2* [16] and AXL [17], mutations in *RAS* [18] and *BRAF* [19], as well as phenotypic alterations [20]. The second‐generation TKIs (e.g., afatinib) irreversibly bind with the four EGFR/ERBB family members. Likewise, a third‐generation TKI, osimertinib, which can inhibit EGFR‐T790M, has been initially approved as a second‐line treatment [21], and it was later approved also as a first‐line treatment [22]. Emergent resistance similarly limits the application of osimertinib. The most common mechanisms of resistance to osimertinib in the first‐line setting are *MET* amplification, emerging C797X mutations, amplification of wild‐type *EGFR* or *HER2*, and mutations in downstream signaling proteins [23].

Following treatment with TKIs, a small subpopulation of NSCLC cells harboring EGFR mutations exhibit reversible tolerance [24, 25]. However, this subpopulation of drug‐tolerant persister cells (DTPs) subsequently acquires irreversible resistance. How DTP states are replaced by permanent drug‐resistant states remains unclear. According to one model, TKI treated cells transiently increase their mutation rates to gain resistance [26]. Unlike the slowly evolving pioneer mutations, which were linked to pre‐existing EGFR mutations in healthy tissues [27], emergence of the secondary (e.g., T790M) and tertiary mutations (e.g., C797S) in patients treated with TKIs is faster and might be adaptive. Recently, two types of mutagenic mechanisms have been reported. One pathway engages apolipoprotein B messenger RNA editing catalytic polypeptide‐like (APOBEC) cytidine deaminases [28, 29, 30, 31]. According to another report, lung cancer cells exposed to TKIs are unable to repair DNA lesions, and they elevate low‐fidelity (mutagenic) DNA polymerases [32]. This second mechanism of induced mutagenesis is preceded by upregulated secretion of GAS6.

GAS6 binds with MERTK, AXL, and TYRO3, a triad of receptor tyrosine kinases that form the TAM family [33]. Rather than functioning as oncogenic drivers, TAM receptors primarily promote stemness and motility. Specifically, MERTK is involved in clearing apoptotic cells and preventing excessive inflammation [34]. The shared ligand, GAS6, also binds phosphatidylserine exposed on apoptotic cells [35]. Unlike AXL, which is a well‐characterized mediator of resistance to TKIs, the role of MERTK in drug resistance has been less defined. Preclinical studies showed that dual targeting of MERTK and EGFR, using the respective TKIs, delayed resistance in a tumor model [36]. Because our re‐analysis of patient data following osimertinib treatment uncovered increased expression of both MERTK and GAS6, we hypothesized that blocking either protein would suppress TKI resistance. By generating anti‐GAS6 and anti‐MERTK antibodies, as well as a bispecific antibody targeting both MERTK and EGFR, we demonstrate that the GAS6–MERTK axis acts as a critical mediator of drug resistance. Blocking this axis using the bispecific EGFRxMERTK antibody sensitizes to TKIs a wide spectrum of mutant forms of EGFR, including not only receptors harboring L858R, T790M, and exon 19 deletions, but also the clinically challenging exon 20 insertions. Importantly, co‐targeting MERTK and EGFR with antibodies achieved greater efficacy than combining their respective TKIs, likely due to the additional contribution of recruiting immune effector cells. Along with mechanistic understanding of the MERTK‐EGFR crosstalk, the antibody‐based tools we have generated may facilitate deeper insights into the GAS6–MERTK signaling axis and set off translation toward clinical application.

## 2 Results

### 2.1 Osimertinib Treatment is Associated With Increased GAS6 Expression in Patients With Residual Disease, and Blocking GAS6 in an Animal Model Can Suppress the Emergence of Drug Resistance

We began by re‐analyzing a single‐cell RNA‐seq dataset from a Phase II osimertinib trial, NCT03433469 (27 patients). This dataset is particularly informative for studying early adaptive responses because drug treatment was relatively short (median, 56 days) and tumor samples were collected immediately before and after treatment [37]. We noted that GAS6 expression was relatively low in treatment‐naïve (TN) tumors but markedly increased in residual disease (RD), accompanied by elevated MERTK and EGFR levels, but AXL abundance remained unchanged (Figure 1A and Figure S1A). These observations suggested that osimertinib‐induced cancer cell death may activate the GAS6–MERTK axis in persister‐like cells within RD, whereas this pathway may become attenuated once tumors progress to overt drug resistance. To test this model, we manipulated GAS6 levels in the PC9 lung cancer cell line expressing an exon 19 mutant of EGFR. Both GAS6 overexpression (OX) and *GAS6* knockout (KO) cells were established. PCR and immunoblotting confirmed the expected alterations in GAS6 and revealed adaptive up‐ and down‐regulation of MERTK and AXL, respectively, in the KO cells (Figure S1B,C). Functionally, GAS6 overexpression enhanced PC9 cell proliferation, whereas GAS6 knockout reduced growth, as measured by crystal violet staining (Figure 1B). Consistently, viability assays demonstrated that GAS6‐overexpressing cells displayed greater resistance to osimertinib, while KO cells were more sensitive to the drug (Figure 1C).

**FIGURE 1:**
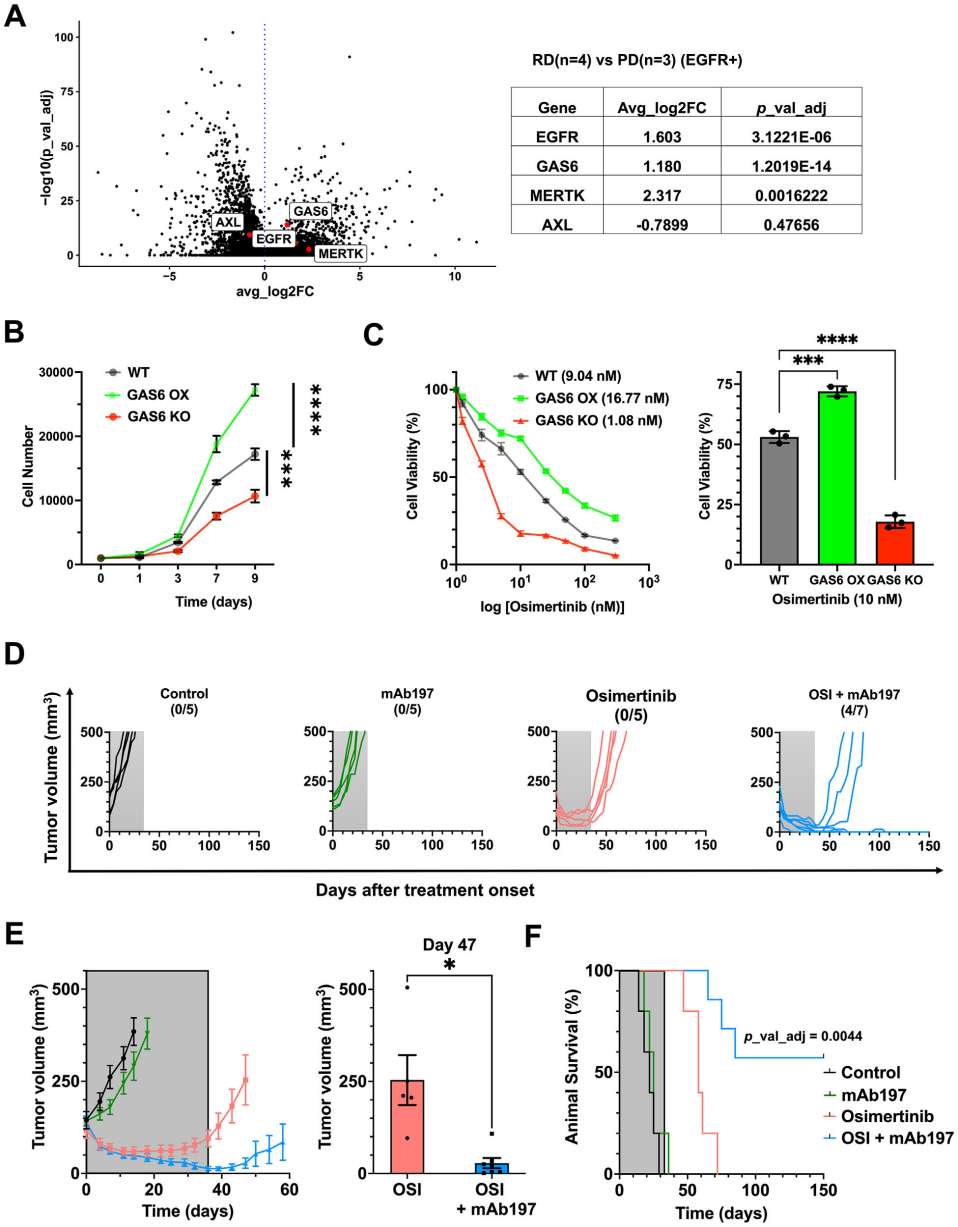
GAS6 supports survival of TKI‐treated cells; blocking it delays resistance to osimertinib in an animal model. (A) Data were taken from a clinical trial, NCT03433469 (EGFR‐positive NSCLC patients). Patients were treated with osimertinib, and the cohort was segregated into Treatment Naïve (TN), Residual Disease (RD), and Progressive Disease (PD). The volcano plot presents delayed early genes that differ between patients with RD (4 samples) and PD (3 samples). MERTK, AXL, GAS6, and EGFR are highlighted in red. The average log2 of the fold change (FC) and the *p*‐values are presented in a table. (B) GAS6 knockout (KO) and GAS6‐overexpressing (OX) PC9 cells were generated. Cells (500/well) were seeded in 96‐well plates and grown for 9 days. The cells were then fixed and stained with crystal violet to estimate cell numbers on specific days. ^***^, *p* < 0.001; ^****^, *p* < 0.0001. (C) The indicated derivatives of PC9 cells (1 × 10^3^, per well) were seeded in 96‐well plates. Next, cells were treated for 72 h with different concentrations of osimertinib. Cell viability was determined using a colorimetric assay. The histogram depicts cell viability under 10 nm osimertinib. (D‐F) PC9 cells (2.5 × 10^6^) were injected subcutaneously in the flanks of CD1‐nu/nu mice. Once the tumor became palpable, 5–7 mice were randomized into the indicated 4 groups and treated for five weeks with either osimertinib (10 mg/kg) or/and a mAb197 against GAS6 (0.2 mg per injection; twice weekly). Individual tumor volumes (D) average tumor volumes per group, along with the average recorded on day 47 (E), and Kaplan‐Meier survival plots (F) are shown. Survival analysis was performed using Holm–Šídák's multiple comparisons test to assess statistical significance between treatment groups. The adjusted *p*‐value for the comparison between osimertinib alone and osimertinib combined with mAb197 is 0.0044. All experiments were repeated at least twice.

Earlier studies showed that GAS6 binds to phosphatidylserine exposed on cells undergoing apoptosis [35] and activates MERTK‐ or AXL‐expressing cycling persisters, likely driving mutagenesis and proliferation of TKI‐resistant cells [32]. Based on this mechanism, we hypothesized that blocking GAS6 might suppress osimertinib resistance. To test this, we generated monoclonal antibodies (mAbs) targeting GAS6. Mice were immunized with a recombinant form of human GAS6, and hybridomas were screened for secretion of anti‐GAS6 antibodies (Figure S1D). The lead clone, 197 (secreting an IgG1 mAb), was selected, and the binding affinity of the mAb was measured using label‐free surface plasmon resonance (SPR). Recombinant GAS6 was immobilized, and the antibody was applied at increasing concentrations. The resulting sensograms (Figure S1E) revealed an equilibrium dissociation constant of 2.3 nm.

Next, PC9 cells were implanted subcutaneously in immunocompromized CD1‐nu/nu mice. Once tumors became palpable, the animals were randomized into four groups (5–7 mice each): untreated control, mice treated with osimertinib alone (administered orally, once daily), mAb197 alone (administered intraperitoneally, twice weekly), and mice receiving the combination of osimertinib and mAb197. Tumor volumes were recorded twice weekly, and body weight was measured once weekly. Treatments were discontinued after five weeks, with no consistent impact observed on body weight (Figure S1F), but tumor growth was monitored for an additional four months. Data are presented as individual tumor growth curves (Figure 1D), average tumor volumes per group (Figure 1E), and Kaplan–Meier survival analysis (Figure 1F). Whereas osimertinib monotherapy resulted in only a temporary delay in tumor regrowth, combining it with the anti‐GAS6 antibody elicited substantially more potent and durable responses, achieving complete tumor regression in 4 of 7 mice, compared with no surviving animals among the five mice treated with osimertinib alone. Collectively, these results identify GAS6 as an important mediator of resistance to a third‐generation EGFR kinase inhibitor.

### 2.2 Endogenous MERTK Supports Resistance of EGFR‐Positive NSCLC Cells to Osimertinib

While the role of AXL in mediating resistance to EGFR inhibitors is well established [32, 38, 39, 40], fewer studies have implicated MERTK in progression and relapse of NSCLC [33, 36, 41]. To investigate MERTK involvement, we made use of a general NSCLC dataset and analyzed patient survival curves of first progression (FP). The curves shown in Figure 2A track the proportion of patients who remained progression‐free at different time points after chemotherapeutic treatments began (note that a different *MERTK* probe was used in each panel). These results indicated that elevated MERTK transcript levels were associated with earlier disease progression. Based on this correlation, we next examined the functional contribution of MERTK to osimertinib tolerance in EGFR‐mutated NSCLC cells, both in vitro and in animals.

**FIGURE 2:**
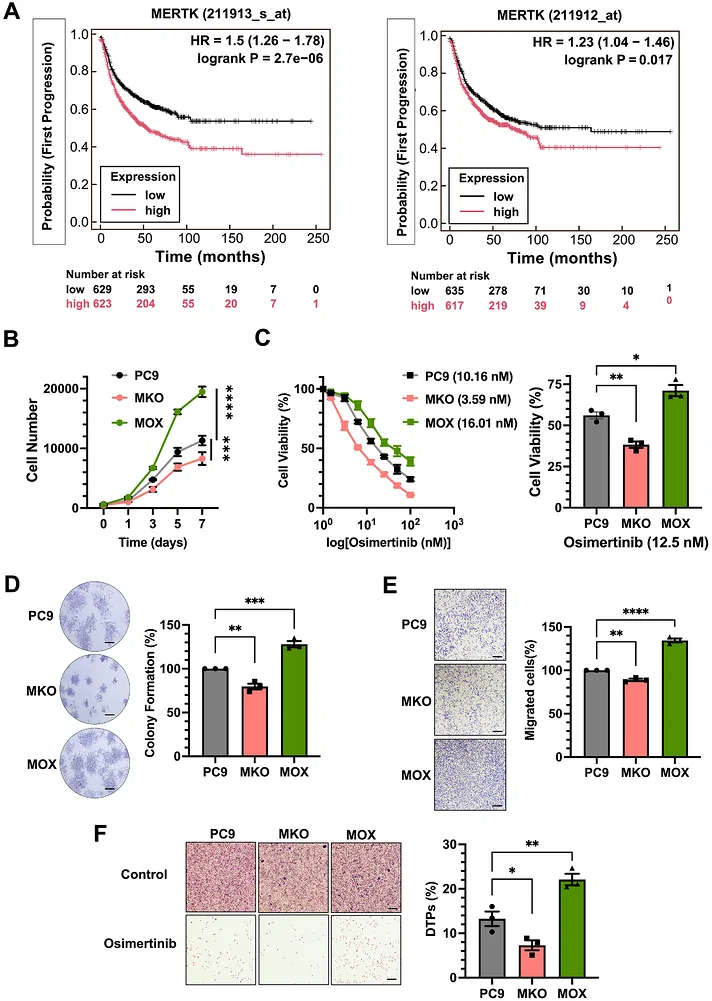
MERTK supports cancer cell survival and enhances resistance to osimertinib. (A) The KM Plotter tool (https://doi.org/10.1111/bph.16257) was used to obtain Kaplan‐Meier survival curves of patients with lung cancer. A dataset of mRNA expression corresponding to 1252 patients was divided into two equal portions according to MERTK levels, high and low, prior to presenting the respective survival curves. Note that each panel shows results obtained with a different oligonucleotide probe. (B) The indicated derivatives of PC9 cells were seeded in 96‐well plates in medium containing 1% fetal bovine serum. Cell growth was measured on the indicated days using crystal violet staining, and the results were presented. The experiment was repeated three times (five technical repeats). ^***^, *p* < 0.001 and ^****^, *p* < 0.0001. PC9, wildtype cells; MKO, MERTK knockout; MOX, MERTK overexpression. (C) The indicated genetically modified and wild‐type PC9 cells (3 × 10^3^ per well) were seeded in 96‐well plates. Next, cells were treated for 72 h with increasing concentrations of osimertinib. Cell viability was determined using the MTT colorimetric assay. The IC50 values were calculated and presented in brackets. The histogram presents cell viability under 12.5 nm osimertinib. ^*^, *p* < 0.05; ^**^, *p* < 0.01. (D) The indicated sublines of PC9 cells (1 × 10^3^ per well) were seeded in 6‐well plates and grown for 9 days. Next, cells were fixed and stained with crystal violet. Representative images of the colonies that were formed are shown (scale bars, 2 mm). The images were scanned and analyzed using ImageJ. ^**^, *p* < 0.01; ^***^, *p* < 0.001. (E) The indicated three PC9 sublines (5 × 10^4^ cells per well) were seeded in transwell chambers in serum‐free medium. The bottom chambers contained media supplemented with serum. Cells were incubated for 18 h. Cells that migrated to the lower face of the wells were fixed and stained using crystal violet. Next, images were taken under a bright‐field microscope. Representative images are shown (scale bar, 2 mm). Quantification of the migrated cells was performed using ImageJ. ^**^, *p* < 0.01; ^****^, *p* < 0.0001. (F) PC9 (wild‐type) and the indicated genetically modified cells were seeded in 6‐well plates at high confluence. Next, they were treated for 9 days with osimertinib (300 nm). The media and drugs were refreshed once every 3 days. Finally, the remaining drug tolerant persister (DTP) cells were fixed and stained with crystal violet. Images were taken from 5 non‐overlapping fields with a light microscope and analyzed using ImageJ. Representative images are shown (scale bar, 0.5 mm). All experiments were repeated at least twice. ^*^, *p* < 0.05; ^**^, *p* < 0.01.

Using the CRISPR technology, we generated PC9 cells lacking endogenous MERTK (MKO cells) and, conversely, engineered PC9 cells to overexpress MERTK via a plasmid construct (MOX cells). Immunoblot analysis of whole‐cell lysates confirmed the anticipated changes in MERTK protein abundance, as well as uncovered adaptive changes in AXL and GAS6 (Figure S2A). Subsequent functional assays demonstrated that MERTK supports proliferation of lung cancer cells: MOX cells showed increased proliferation and expression of Ki67, a proliferation marker (Figure 2B and Figure S2B). In the next step, we tested the possibility that elevated MERTK expression might enhance the tolerance of EGFR+ lung cancer cells to osimertinib. To evaluate this, MKO, MOX, and control PC9 cells were exposed for 3 days to increasing concentrations of osimertinib, followed by assessment of cell viability. As shown in Figure 2C, MERTK overexpression increased the ability of PC9 cells to withstand osimertinib treatment (IC50 increased from 10.16 to 16.0 nm), whereas genetic ablation of *MERTK* clearly sensitized the cells to the EGFR inhibitor (IC50 of 3.59 nm).

Next, we performed a colony formation assay, which confirmed that *MERTK* ablation diminished long‐term proliferative capacity of NSCLC cells harboring a mutant form of EGFR (Figure 2D). Similarly, enhanced migration across porous membranes, another hallmark of aggressive cancer cells, was observed in the MERTK‐overexpressing PC9 derivative (Figure 2E). Given that an earlier study identified a reversible drug‐tolerant state that precedes the irreversible emergence of TKI‐resistant cells [24], we next performed a drug‐tolerant persister assay. Prior to assaying viability, PC9 cells were treated with a relatively high dose of osimertinib for an extended period of time (9 days). This assay revealed that MOX cells generated more drug‐tolerant persister cells compared with MKO and control cells (Figure 2F). Taken together, the results shown in Figure 2 and Figure S2 raised the possibility that adaptive MERTK upregulation may endow lung cancer cells with enhanced tolerance to drug treatment.

Preliminary RT‐qPCR analyses suggested that MERTK loss may trigger compensatory TYRO3 upregulation, whereas AXL loss may increase MERTK and EGFR expression. We therefore investigated the respective contributions of MERTK and AXL to resistance to osimertinib and erlotinib. In similarity to the previously described MERTK‐knockout (MKO) cells, we generated AXL‐knockout (AKO) cells and treated them with erlotinib (50 nm) or osimertinib (10 nm) for 48 h before immunoblot analysis. As shown in Figure S2C,D, MERTK ablation induced compensatory AXL upregulation, whereas AXL ablation increased MERTK and TYRO3, as well as EGFR, HER2, and HER3 expression. Notably, increased EGFR expression was accompanied by enhanced receptor autophosphorylation and ERK activation. These findings reveal extensive compensatory crosstalk between the MERTK receptor family and the EGFR network, prompting us to compare MERTK‐ and AXL‐targeted strategies. To this end, PC9 cells were treated for 72 h with osimertinib (10 nm), the MERTK‐selective TKI MRX‐2843 (300 nm), or the AXL‐selective TKI bemcentinib (100 nm), alone or in dual and triple combinations, followed by cell viability assessment. Under these conditions, no significant differences in viability were detected between the individual treatments and the drug combinations (Figure S2E). This may reflect the potent cytotoxic effects of EGFR‐ and TAM‐family TKIs, which could limit the detection of additional inhibitory effects from combined treatment.

### 2.3 Simultaneous Targeting of EGFR and MERTK Using Monoclonal Antibodies Can Delay the Emergence of Resistance to Osimertinib, Likely by Inhibiting Receptor Crosstalk

Unlike TKIs, mAbs can promote endocytosis and degradation of the respective RTKs. We therefore hypothesized that anti‐MERTK antibodies, in similarity to genetic downregulation of MERTK, would enhance sensitivity to TKIs. To generate such antibodies, mice were immunized with a recombinant extracellular domain of MERTK. Following several rounds of immunization, we obtained evidence of successful immunization (Figure S3A). Therefore, splenic B cells were isolated and fused with a myeloma cell line to generate hybridomas. Screening of the resulting hybridomas (Figure S3B) identified several positive clones, from which one clone (number 745) was selected and expanded. Functional testing of mAb745 in the PC9 xenograft model showed that the antibody alone had no detectable in vivo activity. However, mAb745 markedly improved the therapeutic efficacy of both osimertinib and cetuximab (Figure 3A,B and Figure S3C). For example, none of the mice treated with osimertinib alone survived the study, whereas 2 of 7 animals receiving the combination of mAb745 and osimertinib remained alive. Similarly, only one of 5 mice treated with cetuximab alone survived, compared with 4 of 7 animals treated with the antibody combination.

**FIGURE 3:**
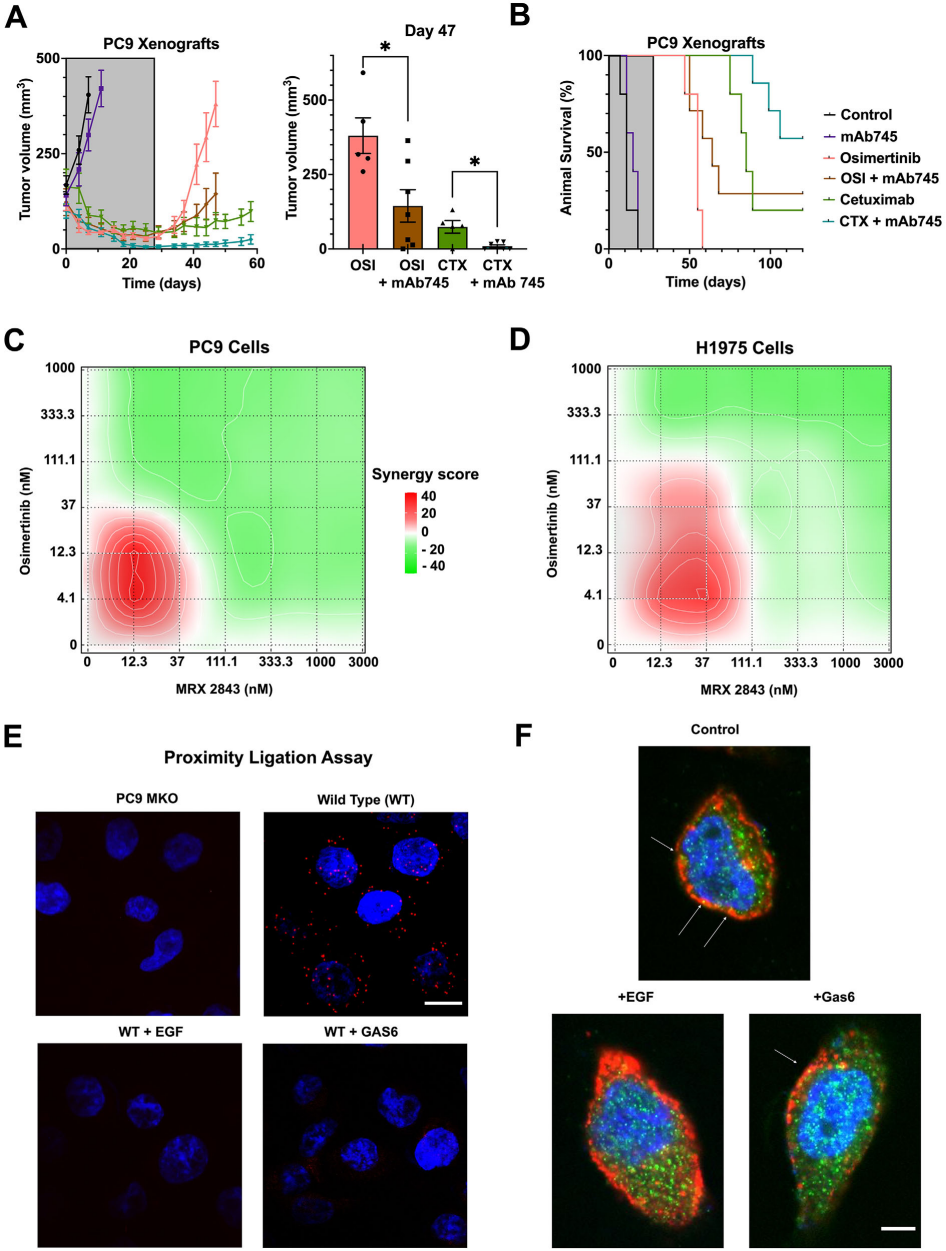
Dual blockade of EGFR and MERTK using antibodies delays tumor relapses, likely due to the ability of EGFR to interact with MERTK within membrane microdomains. (A, B) CD1‐nu/nu mice were subcutaneously implanted with PC9 cells (2.5 × 10^6^). Once tumors became palpable, animals (5–7 per group) were randomized and treated twice weekly for 4 weeks with the indicated mAbs (200 µg/injection, intraperitoneally), and/or daily with osimertinib (5 mg/kg; orally). Tumor volumes were measured twice weekly. Tumor volumes on day 47 are shown as mean ± SEM. Statistical significance was determined using an unpaired *t*‐test; ^*^, *p* < 0.05. Tumor volumes and animal survival are shown. The grey‐shaded area indicates the treatment period. Data are presented as tumor volume (mean ± SEM; A) and the corresponding Kaplan–Meier survival curves (B). (C and D) PC9 and H1975 cells (3 × 10^3^) were seeded in 96‐well plates. The next day, cells were treated for 3 days with different concentrations of osimertinib and MRX‐2843 in pairwise combinations. Following fixation, cells were stained with crystal violet to identify viable cells. The synergy scores of the drugs were calculated based on the Bliss reference model, using SynergyFinder. The red color indicates high synergy scores, implying cooperative EGFR‐MERTK interactions. (E) PC9 cells (1 × 10^4^) were seeded on coverslips and allowed to adhere overnight. The next day, cells were serum starved for 6 h and treated with either EGF (100 ng/mL) or GAS6 (250 ng/mL) in serum‐free medium. Thereafter, the cells were fixed, permeabilized, and then stained with a rabbit anti‐MERTK antibody, as well as with a murine anti‐human EGFR antibody. Subsequently, a proximity ligation assay was performed using anti‐Rabbit Plus and anti‐Mouse Minus. The amplified product of the PLA test was labeled using a red dye, and nuclei were stained with DAPI. Images were obtained using a Zeiss Spinning Disk confocal microscope, and the analysis was performed using ImageJ. MERTK knockout PC9 cells were used as a negative control. Scale bar, 10 µm. All experiments were repeated twice. (F) PC9 cells were seeded on glass coverslips and serum‐starved for 8 h, followed by stimulation for 60 min with either EGF (100 ng/mL) or Gas6 (250 ng/mL). Cells were then fixed and immunostained for EGFR (green) and nuclear DNA (DAPI; blue) and caveolin‐1 (red). Colocalization of EGFR with caveolin‐1 is indicated by white arrows. Scale bar, 5 µm.

To independently confirm that MERTK and EGFR cooperate to sustain the viability of lung cancer cells, we substituted mAb745 with the MERTK‐specific TKI, MRX‐2843, which is currently under clinical development, and evaluated drug interactions using the Bliss synergy model [42]. The assay used two cell lines, PC9 and H1975, which harbor two EGFR mutations, L858R and T790M. Cells were treated for 3 days with increasing concentrations of osimertinib and MRX‐2843 in pairwise combinations. Following fixation, viable cells were stained with crystal violet and drug synergy scores were calculated using SynergyFinder (Figure 3C,D). As indicated by the red color, co‐inhibition of MERTK and EGFR generated high synergy, especially when each drug was present at relatively low concentrations.

Assuming that MERTK and EGFR engage not only in functional but also in physical interactions, we employed the nanoBRET assay [43]. This methodology monitors protein—protein interactions in live cells via bioluminescence resonance energy transfer and has been used by us to characterize the molecular pharmacology of EGFR [44]. As acceptor, the membrane impermeable HaloTag AlexaFluor‐488 substrate was used. The fluorophore labels were attached to the N‐terminus of either HaloTag‐MERTK or HaloTag‐EGFR. When the donor and acceptor are in proximity (0–10 nm), NanoLuc emission excites the fluorophore on the HaloTag sequence, which then emits light at a longer wavelength (schematize in Figure S3D). Consistent with spontaneous EGFR homodimerization, we observed significant BRET signals when NanoLuc and HaloTag were separately fused to EGFR and co‐expressed (Figure S3E). In contrast, no evidence was obtained for MERTK homodimers or for MERTK‐EGFR heterodimerization.

Presuming that MERTK‐EGFR inter‐receptor distances exceed the < 10 nm distance requirement for efficient BRET, we used the proximity ligation assay (PLA), which relies on pairs of antibodies and detects interactions within an effective range of ∼ 10–40 nm. Gratifyingly, PLA revealed numerous EGFR–MERTK clusters in PC9 cells, whereas no signal was observed in MERTK‐knockout cells, confirming assay specificity (Figure 3E). Impressively, pretreating naïve PC9 cells with either GAS6 or EGF almost completely abolished the PLA signal, consistent with a previously described mechanism that concentrates unstimulated RTKs within caveolae [45]. According to this and additional reports, following ligand binding, several RTKs, including EGFR, rapidly exit caveolae in a process coupled to receptor auto‐phosphorylation and SRC kinase activation. To examine relevance to NSCLC, we stimulated for 60 min serum‐starved PC9 cells with either EGF or GAS6 and then fixed them. Subsequent immunofluorescence analysis detected in control cells many puncta containing caveolin‐1 and EGFR, but much fewer co‐localization puncta were detected in cells preincubated with either GAS6 or EGF (Figure 3F). Taken together, these findings imply that the ability of the anti‐MERTK antibody to enhance the efficacy of osimertinib and cetuximab might be mediated by disrupting receptor crosstalk.

### 2.4 A Bispecific MERTKxEGFR Antibody Enhances Endocytosis and Degradation of Both MERTK and EGFR

Bispecific antibodies expand the therapeutic arsenal beyond monoclonals by dual receptor blockade and combining tumor targeting with immune activation [46]. Compared with conventional mAbs, bsAbs can block compensatory signaling pathways, promote receptor internalization and degradation, and increase specificity by requiring co‐recognition of two tumor‐associated antigens. For the design of the bispecific anti‐EGFRxMERTK antibody (termed Bis‐EM; see a schematic diagram in Figure 4A and amino acid sequences of all four immunoglobulin chains under Methods), we replaced the murine Fc region of mAb745 and combined the recombinant forms of mAb745 and cetuximab (an anti‐EGFR mAb), both carrying a human Fc domain. To prevent mispairing of the light and heavy chains during co‐expression of the two antigen‐binding arms, the EGFR‐binding arm of cetuximab was reformatted as a CrossMab with a CH1–CL domain swap [47]. Additionally, heterodimerization of the two heavy chains was enforced through the following CH3 knobs‐into‐holes mutations [48]: Y349C/T366S/L368A/Y407V in the EGFR arm and S354C/T366W in the MERTK arm. Binding affinities of Bis‐EM and its parent molecules toward recombinant forms of MERTK and EGFR were assessed using SPR (Figure S4A–D). The calculated dissociation constants (Kd), derived from the ratio of the dissociation rate constant (koff) to the association rate constant (kon), are summarized in Figure 4A. As shown, Bis‐EM displayed an approximately fourfold lower affinity for MERTK relative to the parental recombinant antibody, while its affinity for EGFR was substantially reduced relative to cetuximab. This decrease in binding strength of both antigen‐binding arms is not uncommon and attributable to altered protein folding. Importantly, the results of a sandwich ELISA test that used an immobilized MERTK were consistent with simultaneous engagement of both MERTK and EGFR molecules by Bis‐EM (Figure S4E). In addition, we confirmed that Bis‐EM was able to precipitate both EGFR and MERTK, as expected from a bivalent molecule having dual antigen specificity (Figure S4F).

**FIGURE 4:**
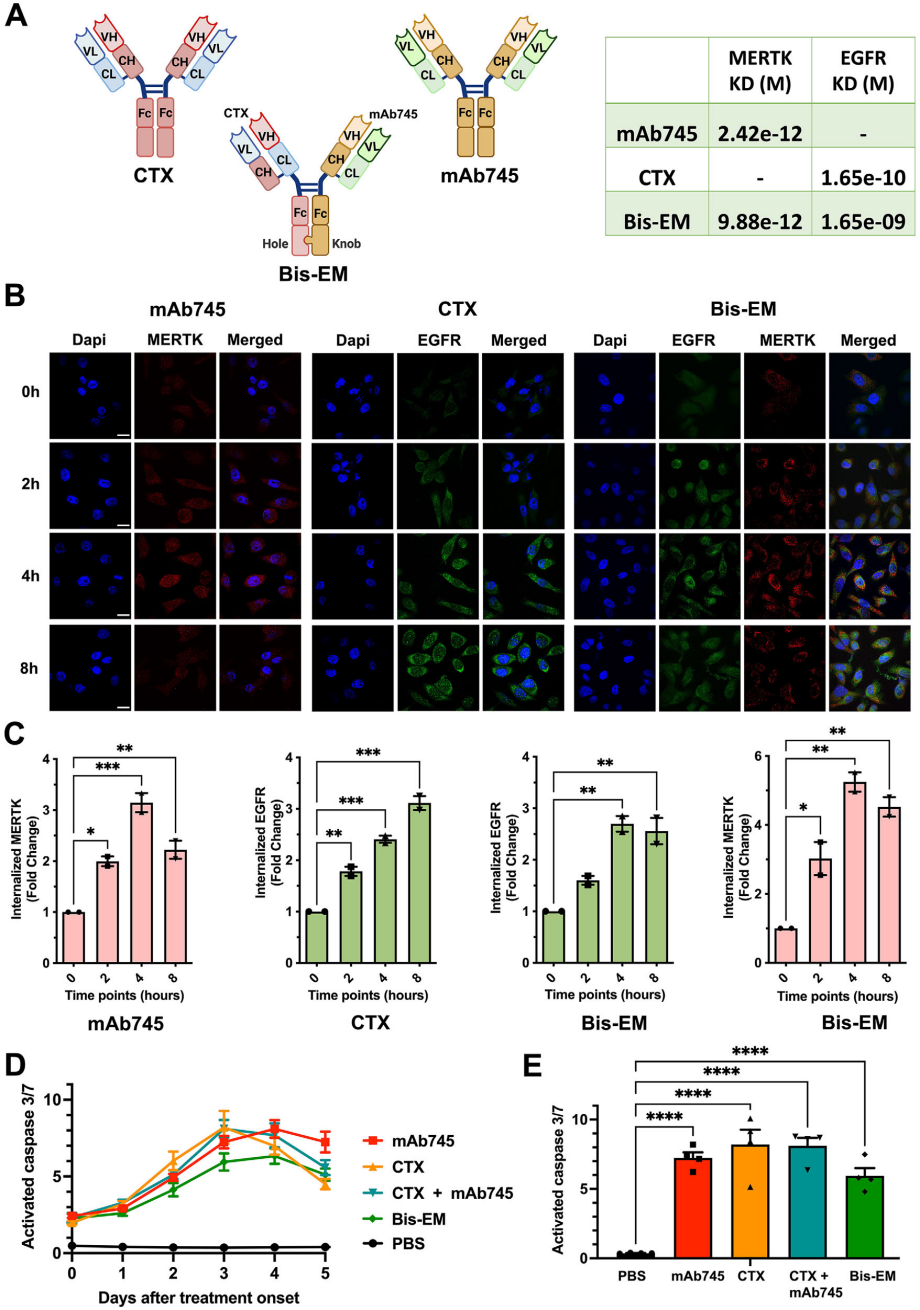
Design and in vitro activities of the anti‐MERTK/EGFR bispecific antibody (Bis‐EM). (A) Schematic representation of the bispecific antibody, Bis‐EM, and the parental antibodies, anti‐MERTK mAb745 and cetuximab. The EGFR‐binding arm was formatted as a CrossMab (CH1–CL swap) to ensure correct heavy–light chain pairing. Additionally, heterodimerization of the heavy chains was achieved through inserting the following mutations in the CH3 domain: Y349C, T366S, L368A, and Y407V in the EGFR arm, as well as two mutations, S354C and T366W, in the MERTK arm. V denotes an antibody variable domain, and C marks a constant antibody domain. Likewise, H denotes a heavy antibody chain, and L denotes the light chain. The table lists parameters obtained using Surface Plasmon Resonance (SPR), which determined binding affinities (dissociation constant, Kd) of the parental antibodies, as well as the Kd of Bis‐EM, toward recombinant soluble forms of human MERTK and human EGFR. CTX, cetuximab. This scheme was made using BioRender. (B, C) PC9 cells (5 × 10^4^) were seeded on coverslips. The next day, cells were treated for various time intervals with the following antibodies: CTX (cetuximab; 10 µg/mL), mAb745 (10 µg/mL), and Bis‐EM (20 µg/mL). Next, cells were fixed (with 4% PFA), permeabilized, and stained with anti‐MERTK and anti‐EGFR antibodies, which were followed by fluorescently tagged secondary antibodies. DAPI was used to visualize nuclei. Images were taken using a spinning disk confocal microscope, and a series of Z‐stacks were taken with a Z‐step of 1 µm. To quantify the intracellular levels of the indicated receptors, the middle section of each cell was selected from the Z‐stack. For each channel, we calculated the average signal in the cytosol (see histograms in C). The obtained signal intensity was used as a threshold value to remove background cytosolic signals. The spots were counted using the function “analyze particles” of ImageJ. The signal corresponding to receptor internalization at t = 0 was used for normalization. ^*^, *p* < 0.05; ^**^, *p* < 0.01; ^***^, *p* < 0.001. Scale bar, 15 µm. All experiments were repeated at least twice. (D, E) PC9 cells (3 × 10^3^ cells/well) were seeded in 96‐well ultra‐low‐attachment plates and cultured overnight to generate spheroids. Spheroids were treated with the indicated mAbs (10 µg/mL) or with the bispecific antibody Bis‐EM (20 µg/mL) and cocultured with human PBMC at an effector‐to‐target ratio of 50:1. Plates were imaged continuously using the IncuCyte system. Caspase‐3/7 activation was quantified by monitoring fluorescence intensity over 5 days (D). Statistical comparisons between treatment groups were based on fluorescence values measured on day 3 (E). ^****^, *P* < 0.0001.

Typically, mAbs directed against cell‐surface receptors can trigger receptor internalization and endocytosis [49]. Clinically, this attribute might be important: internalization can attenuate receptor signaling, but excessive uptake may reduce receptor availability for potential antibody‐dependent cellular cytotoxicity (ADCC). To address receptor endocytosis and downstream signaling, we exposed PC9 cells to increasing antibody concentrations (Figure S4G) or to extended treatment interval (Figure S4H). Immunoblotting analysis validated downregulation of MERTK and EGFR by the respective antibody, as well as downregulation of both receptors following Bis‐EM treatment (see protein band quantification underneath each lane of Figure S4G,H). Consistent with receptor crosstalk, we also observed less extensive downregulation of EGFR by mAb745 and vice versa, MERTK by cetuximab. In addition, we observed consistent antibody‐induced downregulation of the ERK pathway but less uniform decreases in the phospho‐AKT signals.

To further examine antibody‐induced receptor internalization, we employed immunofluorescence. Cells were treated with Bis‐EM or with the parental antibodies, fixed, and probed using the respective polyclonal antibodies. Critically, imaging was performed using a spinning disk confocal microscope that was focused beneath the plasma membrane in a way that captured internalized receptors. As shown in Figure 4B and quantified in the accompanying histograms (Figure 4C), both MERTK and EGFR underwent internalization within 2–4 h of treatment with the respective antibody and, as anticipated, Bis‐EM promoted internalization of both receptors. In summary, Bis‐EM retained significant binding affinities for both EGFR and MERTK and could drive their internalization and intracellular degradation.

### 2.5 The Bispecific Bis‐EM Antibody Can Increase Cleavage of Caspases, As Well As Enhance Perforin Secretion in the Presence of Immune Cells

We next examined the ability of Bis‐EM to induce antibody‐mediated immune cell killing by promoting tumor cell apoptosis and increasing the release of cytotoxic mediators, such as perforin, when immune effector cells are present. To this end, we generated spheroids of PC9 cells and exposed them to Bis‐EM, or to the parental antibodies, in the presence of human peripheral blood mononuclear cells (PBMCs). Using the Incucyte imaging system, the co‐cultures were continuously monitored for five days for cleavage of caspase‐3 and caspase‐7, a hallmark of apoptosis. The results showed that Bis‐EM induced a pattern of apoptosis comparable to that observed with its parental antibodies (Figure 4D,E and Figure S4I). In line with time‐dependent antibody‐mediated immune killing, co‐culturing PC9 cells and PBMC in the absence of antibody displayed no caspase‐3/7 cleavage. Moreover, ELISA measurements detected increased perforin release (Figure S4J), reflecting activation of effector cells through Fc‐receptor engagement. In summary, Bis‐EM demonstrated both the ability to accelerate degradation of its two target receptors and the capacity to promote immune‐mediated tumor cell killing in the presence of blood cells.

### 2.6 Concurrent Inhibition of MERTK and EGFR, Using Either Bis‐EM or a Combination of the Respective TKIs, Comparably Suppresses Growth in 2D and 3D NSCLC Models

Potentially, combining two TKIs, each specific to one receptor, might serve as an alternative to the use bispecific antibodies like Bis‐EM. As a prelude to in vivo comparisons of Bis‐EM and TKI combinations, we performed colony formation assays using PC9 (harboring an EGFR exon 19 deletion) and H1975 cells harboring the EGFR T790M mutation along with L858R (Figure 5A). Cells were treated for nine days with osimertinib, MRX‐2843, Bis‐EM, or the parental anti‐EGFR and anti‐MERTK antibodies. Quantification of colonies showed that the triplet combination MRX‐2843 + osimertinib + cetuximab achieved growth inhibition comparable to that of the Bis‐EM + osimertinib doublet or the respective triplet regimen (Figure 5A). Consistent with these findings, the same combinations were the most effective inhibitors in drug‐tolerant persister assays (Figure 5B), which measured the ability of treatments to suppress formation of reversible persister cells able to seed stable resistance. A short‐term viability assay after three days of treatment (Figure S5A) further confirmed that dual blockade of EGFR (osimertinib + cetuximab) and MERTK (Bis‐EM, mAb745, or MRX‐2843) was required for maximal suppression of cell survival in our in vitro models.

**FIGURE 5:**
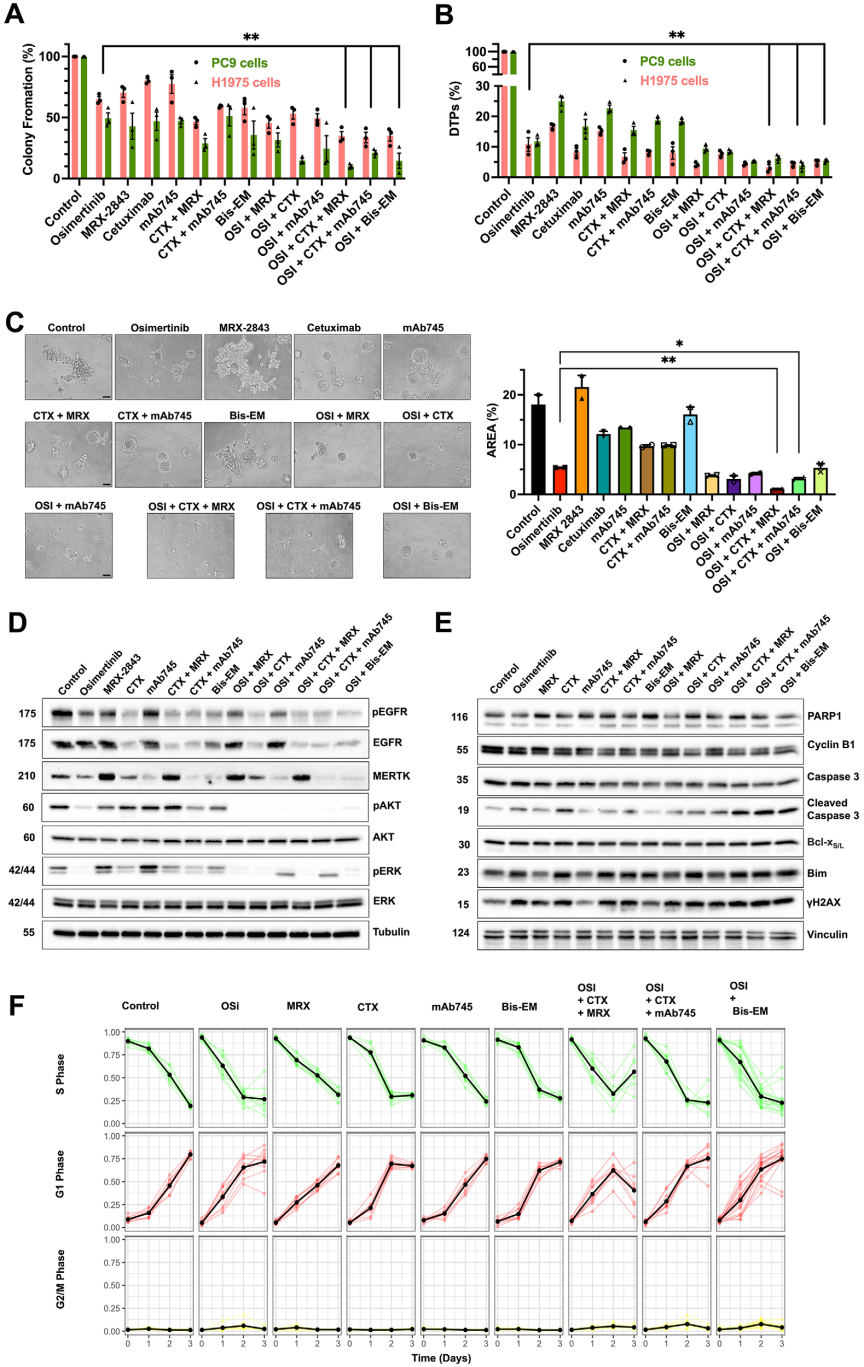
Simultaneous inhibition of MERTK and EGFR suppresses growth of NSCLC cells and associates with both inhibition of signaling and activation of apoptosis. (A) PC9 (EGFR exon 19 deletion) and H1975 cells (EGFR‐L858R in exon 21 and the “gatekeeper” mutation T790M) were seeded at low density in 6‐well plates (1000 cells/well). The next day, cells were treated for 9 days with anti‐EGFR (cetuximab), anti‐MERTK mAb (mAb745; each at 10 µg/mL), Bis‐EM (20 µg/mL), osimertinib (10 nm), or MRX‐2843 (300 nm). The drugs were replenished once every 72 h. Next, cells were fixed and stained with crystal violet. Images were captured using a bright‐field microscope. Colonies were quantified (CFU, colony formation units) using ImageJ. The experiment was repeated thrice. (B) PC9 and H1975 cells were seeded in 6‐well plates (5 × 10^5^ cells per well). Both assays were performed in 6‐well plates. The next day, cells were treated for 9 days as in A, except that osimertinib was used at 300 nm and MRX‐2843 at 1000 nm. Media were replenished once every 3 days. Next, they were fixed and stained with crystal violet. Images of surviving cells were captured using a bright‐field microscope. Drug tolerant persister (DTP) cells were quantified using ImageJ. (C) PC9 cells (1 × 10^3^) embedded in 5% BME were seeded in BME pre‐coated 96‐well plates. The wells contained vehicle control (CTR), mAbs (10 µg/mL), osimertinib (10 nm), MRX‐2843 (300 nm), Bis‐EM (20 µg/mL), and the indicated drug combinations. All experiments were stopped after 7 days. Images of the formed spheroids were captured using an OpTech IB4 microscope (bright field; 20x magnification). Percentages of the substrate covered areas were assessed using ImageJ. Significance was calculated using one‐way ANOVA, Dunnett's and Tukey's multiple comparison tests (^*^, *p* < 0.05; ^**^, *p* < 0.01). The histograms represent the average of two independent experiments performed in duplicates. Scale bar, 50 µm. (D, E) H1975 cells were grown to confluence in 10‐cm dishes and treated for 48 h with the indicated TKIs and antibodies, either alone or in combination, at the concentrations indicated in A. Cell lysates were analyzed using immunoblotting and the indicated antibodies. Tubulin and vinculin served as loading controls. The left panel shows downstream signaling, and the right panel shows apoptosis‐related signaling. Representative blots from one of two independent experiments are shown. (F) PC9 cells expressing the Fucci reporter were treated for 1–3 days with the indicated agents or their combinations at the following concentrations: osimertinib (10 nm), MRX‐2843 (300 nm), cetuximab (10 µg/mL), mAb745 (10 µg/mL), and Bis‐EM (20 µg/mL). Images were collected daily from at least nine distinct fields of view. Nuclei were detected based on nuclear Fucci fluorescence, and valid nuclei were selected using circularity thresholds to remove fluorescent debris. Green and red signal intensities were measured to assign cells to S phase, G1 phase, or G2/M. Image acquisition was performed on a CV8000 system, followed by processing that made use of Fiji, segmentation, quantification with CellProfiler, and data visualization in R Studio. Note: Original blots corresponding to panels (D, E) can be found in Figure S8A.

Unlike 2D monolayers, tumor resistance to anti‐cancer drugs frequently associates with diffusion barriers and tumor–matrix interactions, both of which are more accurately preserved in 3D spheroids. These multicellular aggregates recapitulate key features of tumors, thereby providing a physiologically relevant context for investigating mechanisms of drug resistance [50]. Hence, we established 3D cultures of PC9 cells embedded in basement membrane extract (BME), a matrix‐rich environment containing proteins such as collagens and laminin and treated them for seven days with various drug combinations. Notably, the triplet combination of osimertinib, cetuximab, and MRX‐2843 consistently produced slightly stronger inhibition than regimens in which MERTK was blocked with an antibody (Figure 5C). Conceivably, this superiority reflects the influence of spheroid architecture and diffusion gradients unique to each drug.

### 2.7 Simultaneous Inhibition of MERTK and EGFR Suppresses Growth of an Osimertinib‐Resistant Model, Likely by Inducing Cell Cycle Arrest and Apoptosis

To extend our observations to an osimertinib‐resistant model, we utilized PC9‐AZDR cells, a derivative line that had evolved resistance to osimertinib following prolonged exposure to the drug (> 3 months). As expected, spheroids generated from PC9‐AZDR cells were largely unresponsive to osimertinib (Figure S5B,C). Nevertheless, PC9‐AZDR cells displayed sensitivity to the triplet regimen (osimertinib + cetuximab + MRX‐2843) and to the doublet combo (osimertinib + Bis‐EM). Although the PC9‐AZDR model, like the other models, demonstrated that concurrent blockade of EGFR and MERTK is necessary for maximal suppression of NSCLC cell survival, the underlying biochemical mechanisms remained unresolved. To address these mechanisms, we made use of antibodies specific to either signaling proteins (Figure 5D) or a set comprising cell cycle‐ and apoptosis‐controlling antigens (Figure 5E). These analyses revealed that the drug triplet (osimertinib/cetuximab/MRX‐2843), like the doublet of drugs (osimertinib/Bis‐EM), robustly inhibited the ERK and AKT pathways while sorting EGFR for degradation, but only the drug doublet accelerated degradation of MERTK. Nevertheless, both treatments activated caspase‐3 cleavage, increased phosphorylation of histone 2X (H2AX), and induced BIM, a pro‐apoptotic BCL‐2 family member that is essential for apoptosis induced by EGFR‐specific TKIs [51].

Along with inhibition of ERK, a known regulator of cyclin D, co‐targeting MERTK and EGFR reduced the expression of cyclin B1 (Figure 5E), a key positive regulator of cell‐cycle progression that controls the transition from G2 to mitosis. To more directly examine cell‐cycle control, we employed the FUCCI system, which is often considered superior to flow cytometry or BrdU incorporation, because it enables dynamic visualization of cell‐cycle phases in individual cells [52]. PC9 cells were first infected with FUCCI‐expressing lentiviruses and then treated with the indicated drug combinations for 1–3 days. High‐content imaging was subsequently used to quantify fluorescence intensities and determine the fractions of proliferating cells (S phase), arrested cells (G1 phase), and cells in G2/M. Population averages were calculated using CellProfiler [53] and are shown in Figure 5F. As illustrated, treatment with TKIs produced a more pronounced reduction in S‐phase cells and a parallel increase in G1 arrest compared with antibody‐based treatments. Notably, the triplet regimen containing MRX‐2843 moderately increased the S‐phase fraction on day 3, a pattern suggesting the emergence of drug‐tolerant cells. Overall, across multiple methodologies, three cellular models, and both 2D and 3D culture systems, we learned that co‐inhibition of EGFR and MERTK produces stronger inhibitory effects than targeting EGFR alone. However, in vitro comparisons between Bis‐EM and the combination of either two TKIs or two mAbs revealed no major difference in terms of the efficacy of growth inhibition.

### 2.8 Combined Bis‐EM and Osimertinib Treatment Durably Suppresses Xenografts but the Triplet osimertinib + cetuximab + mAb745 was More Effective than the osimertinib + cetuximab + MRX Triplet

To assess whether dual inhibition of EGFR and MERTK can control tumor growth in an animal model, we generated xenografts by subcutaneously implanting either PC9 cells (exon 19) or H1975 cells (exons 20 and 21) in the flanks of immunocompromized mice. Once tumors became palpable, animals were randomized into treatment groups. Small‐molecule inhibitors (osimertinib and MRX‐2843) were administered orally once daily, whereas antibodies (Bis‐EM, cetuximab and mAb745) were delivered intraperitoneally twice weekly. Treatments were discontinued after five weeks, but tumor growth and body weight were monitored for an additional four months. Outcomes are presented as Kaplan–Meier survival plots (Figure S6A,B,C,D), and individual tumor growth curves (Figure 6A,B). No consistent weight loss, used as a surrogate for systemic toxicity, was observed across treatment groups (see below). In contrast, tumor burden and survival varied substantially. As expected, osimertinib monotherapy initially reduced tumor size, but relapses occurred soon after treatment cessation. In general, combination regimens yielded stronger and more durable effects: all osimertinib‐based doublets extended survival compared with osimertinib alone, with osimertinib + cetuximab being an effective pair. Importantly, the adjusted *p*‐value for the comparison between osimertinib alone and osimertinib combined with Bis‐EM was 0.0013. Notably, unlike the in vitro results, the triplet osimertinib + cetuximab + mAb745 was more effective than the MRX‐2843–containing triplet. Furthermore, the Bis‐EM + osimertinib doublet achieved efficacy comparable to that of the osimertinib + cetuximab + mAb745 triplet across both xenograft models.

**FIGURE 6:**
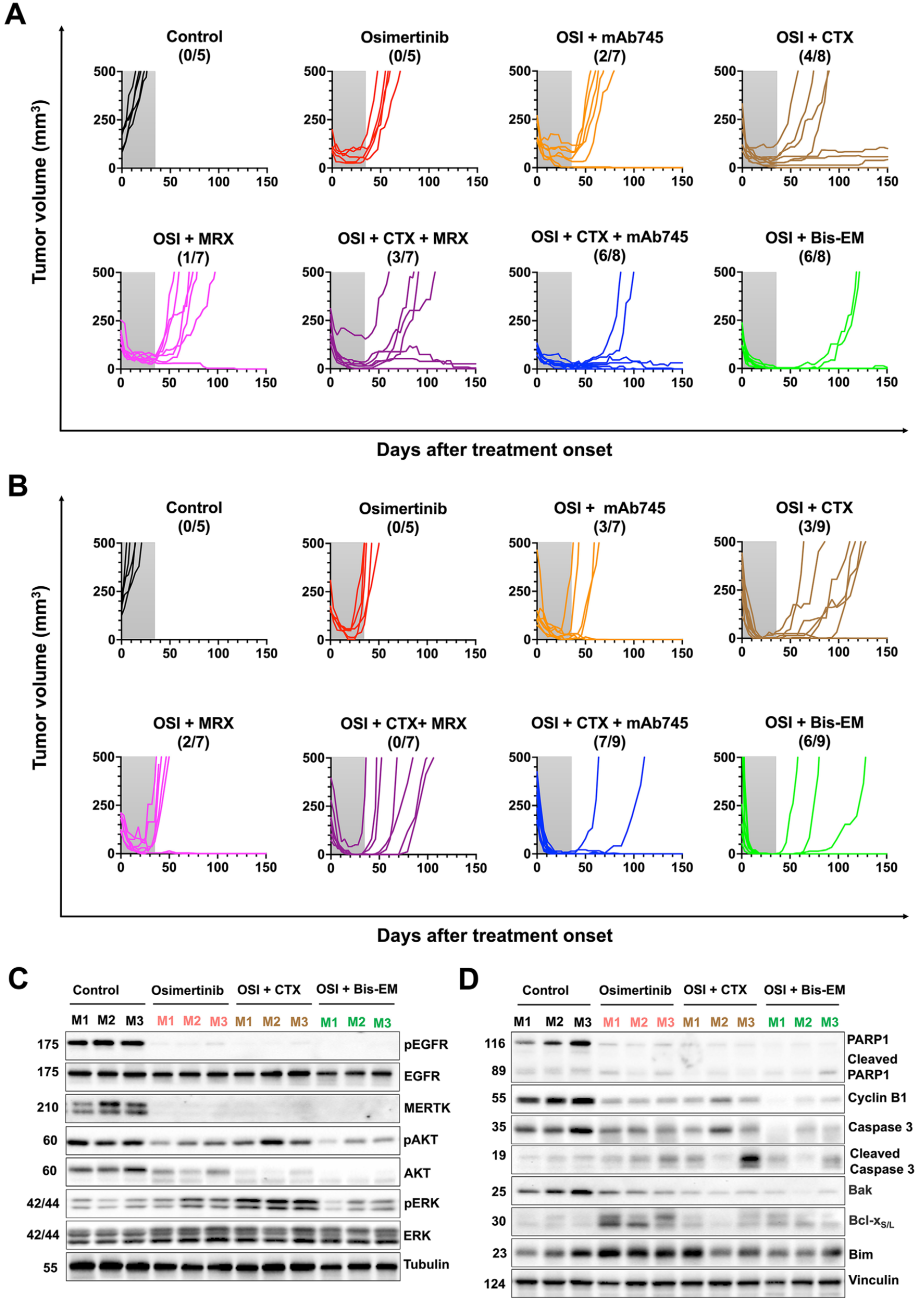
The in vivo efficacy of the combination of Bis‐EM plus osimertinib surpasses that of osimertinib+cetuximab+MRX‐2843. (A) PC9 cells (2.5 × 10^6^ per animal) were injected subcutaneously in the flanks of CD1‐nu/nu mice. Once tumors became palpable, 5–9 mice were randomized and treated for five weeks with the indicated agents, either mAbs (100 µg/injection) or Bis‐EM (200 µg/injection), twice a week. In addition, where indicated, we used osimertinib (10 mg/kg) or MRX‐2843 (20 mg/kg), which were orally administered once per day. Tumor volumes were monitored twice a week, and animal body weight was determined once per week. Note that each line represents one animal. The number of mice surviving at the end of the experiment is shown in parentheses for each group. Shaded areas mark the drug treatment phase. Note that the Control and the Osimertinib panels also appear in Figure 1D. (B) The H1975 xenograft model was used, and the results are presented essentially as described for the PC9 model in A. (C and D) PC9 cells (2.5 × 10^6^ cells/mouse) were subcutaneously injected into the right flanks of 6‐week‐old CD1 nude mice. When tumors became palpable, mice were randomized into four treatment groups (3–4 mice per group) and treated with osimertinib (10 mg/kg, oral gavage) and/or the indicated antibodies (cetuximab, 0.1 mg/mouse per injection; Bis‐EM, 0.2 mg/mouse per injection). Antibodies were administered twice weekly by means of intraperitoneal injection. Tumor volumes were measured during treatment. After 7 days, tumors were excised and processed for protein extraction. Tumor lysates were subjected to gel electrophoresis and immunoblotting, with vinculin and tubulin used as loading controls. Panel C depicts effects on EGFR‐dependent downstream signaling, whereas panel D depicts the status of apoptosis and cell cycle related pathways. Each lane corresponds to an individual mouse. Note: Original blots corresponding to panels (C, D) can be found in Figure S8A.

Because a bispecific EGFRxAXL antibody (Bis‐EA) we previously constructed effectively delayed the onset of resistance to osimertinib in animal models [40], it was interesting comparing its efficacy to that of Bis‐EM. Using the H1975 xenograft model and CD1‐nu/nu mice, we noted that simultaneous targeting of EGFR and either AXL or MERTK provided highly similar protection against tumor relapses (Figure S6E,F). As predicted, combinations of the respective monoclonal antibodies were as effective as the corresponding bispecific antibodies. These findings further support the therapeutic potential and robustness of simultaneously targeting EGFR together with either MERTK or AXL. To investigate the molecular mechanisms underlying the ability of the Bis‐EM + osimertinib regimen to prevent or markedly delay tumor relapse, we repeated the PC9 xenograft experiment using 3–4 mice per treatment arm. Animals were treated, or left untreated, for only seven days prior to tumor excision. Figure S6G,H shows the growth curves and relative tumor volumes at the time of tumor collection.

Immunoblot analysis of tumor lysates (Figure 6C,D) revealed that, in parallel to tumor regression under treatment, all drug‐treated groups displayed loss of both pEGFR and MERTK signals. However, downregulation of total EGFR protein was observed only in tumors from mice receiving the Bis‐EM + osimertinib combination. Consistent with the pronounced tumor regression seen in this group, the respective tumors also showed near‐complete loss of pAKT and pERK, along with markedly reduced levels of PARP1, caspase‐3, cyclin B1, and BIM. In summary, the bispecific antibody combined with osimertinib recapitulated the potent and durable tumor control observed with the most effective triplet regimen (osimertinib + cetuximab + mAb745). This superior performance of the anti‐MERTK antibody relative to the MERTK kinase inhibitor in combination treatments may reflect its slower systemic clearance or the ability of antibodies, unlike TKIs, to recruit anti‐tumor immune effector cells.

### 2.9 The Bispecific EGFRxMERTK Antibody Prolongs Remissions in PDX Models Harboring EGFR Mutations in Either Exon 19 or Exon 20

Patient‐derived xenograft (PDX) models maintain key molecular characteristics of the original tumors, including intratumoral heterogeneity, and therefore more accurately reflect drug resistance mechanisms than traditional cell line xenografts [54]. To test Bis‐EM in an exon 19 PDX model, we selected the TM00193 model (E746_A750 Del19‐EGFR, from The Jackson Laboratory) and subcutaneously implanted fragments in NSG immunocompromized mice. Once tumors reached ∼ 350 mm^3^, animals were randomized into treatment groups and received osimertinib, recombinant mAbs, or Bis‐EM for a four‐week period. Tumor growth and animal survival were monitored for up to 120 additional days (Figure 7A,B and Figure S7A). The data showed that Bis‐EM worked synergistically with osimertinib, preventing the typical rapid relapse observed after drug withdrawal. Moreover, consistent with the cell line xenograft studies, Bis‐EM produced efficacy comparable to the respective triplet combination (osimertinib + cetuximab + mAb745).

**FIGURE 7:**
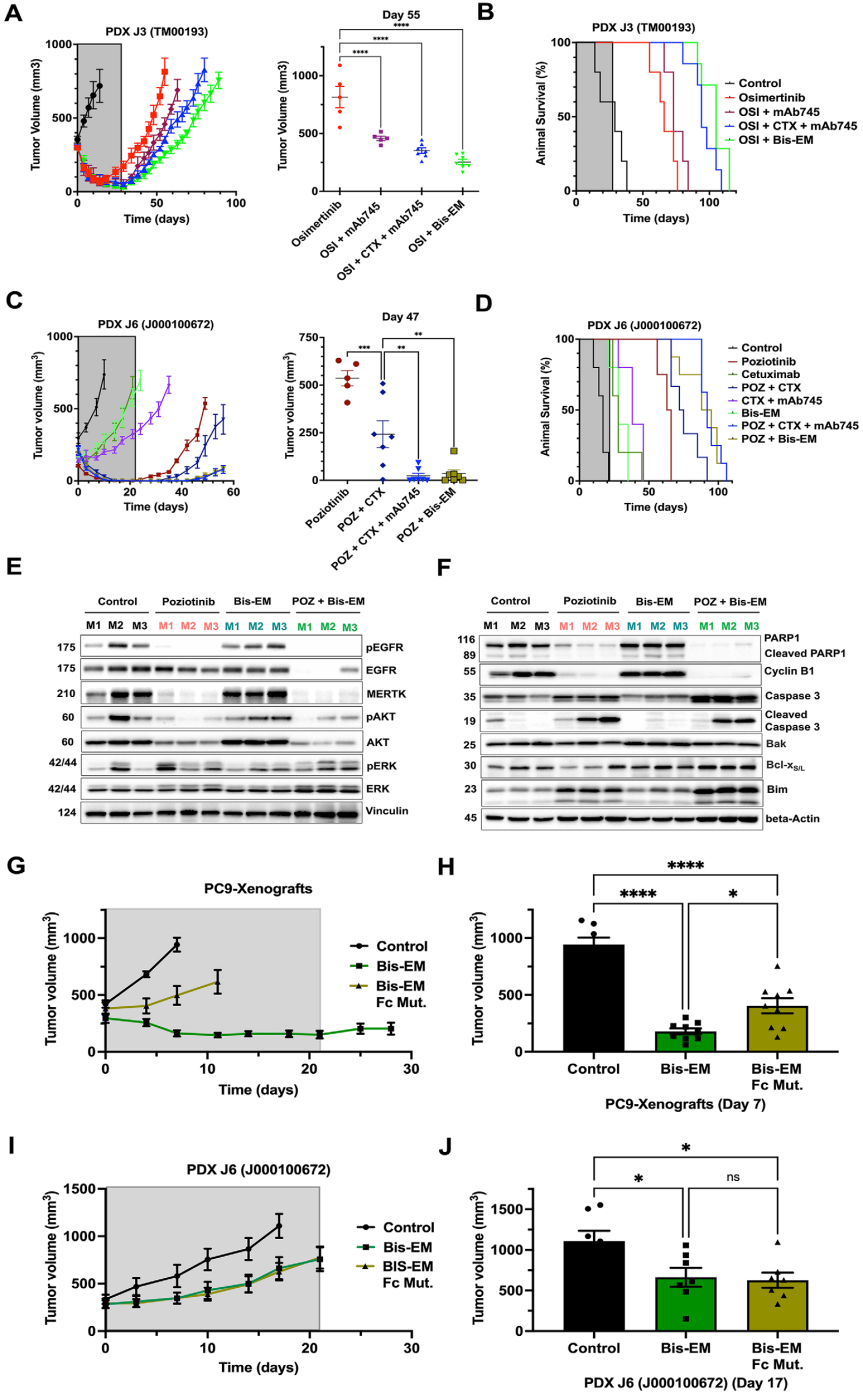
The EGFRxMERTK bispecific antibody combined with a TKI delays relapse in PDX models in an Fc‐dependent manner. (A, B) The patient‐derived xenograft TM00193 (E746_A750 Del19‐EGFR) was engrafted subcutaneously in NSG mice. When tumors became palpable (around 350 mm^3^), mice were randomized in groups of 5–7 animals and treated for four weeks (shaded areas) with osimertinib (10 mg/kg/day; orally) and the indicated mAbs (100 micrograms/mouse/injection), or with Bis‐EM (200 micrograms/mouse/injection). Tumor volume and animal body weight were monitored for the next 90 days. The average tumor volume of each group is presented both longitudinally and on Day 55. Data shown are means ± SEM. Kaplan–Meier survival curves corresponding to each experimental arm are presented in B. Survival analysis was performed using Holm–Šídák's multiple comparisons test to assess statistical significance between treatment groups. The adjusted *p*‐value for the comparison between osimertinib alone and osimertinib combined with Bis‐EM is smaller than 0.001. (C, D) NSG mice were implanted with a lung cancer PDX model, PDXJ6 (from The Jackson Laboratory, J000100672), harboring the exon 20 mutations S768_V769insSVD. Once tumors reached approximately 300 mm^3^, mice were randomized into groups of 5–7 animals per group. They were later treated for 3 weeks (shaded areas), twice a week, with the indicated mAbs (100 µg of each mAb/injection) or with Bis‐EM (200 µg/injection). Alternatively, mice were treated with poziotinib (5 mg/kg), an exon 20–specific TKI that was administered orally once daily. Tumor growth and body weights were monitored twice per week. Shown are the average tumor volumes, including day 47 statistics, and animal survival times. Data shown are means ± SEM. Survival analysis was performed as in (B). The adjusted *p*‐value for the comparison between POZ+CTX and POZ+CTX+mAb745 is smaller than 0.01. Similarly, the adjusted *p*‐value for the comparison between POZ+CTX and POZ + Bis‐EM is less than 0.05. POZ, poziotinib; CTX, cetuximab. (E, F) NSG mice were implanted subcutaneously with fragments derived from the PDX model harboring an EGFR exon 20 mutation S768_V769insSVD. Once tumors reached approximately 300 mm^3^, mice were randomized into treatment groups (3 mice per group; M1 through M3). Next, mice were treated for 7 days with Bis‐EM (250 µg per injection), which was administered twice weekly. Where indicated, mice received the exon 20–selective tyrosine kinase inhibitor poziotinib (POZ; 5 mg/kg). This TKI was administered orally once daily, either alone or in combination with Bis‐EM. Seven days after treatment initiation, tumors were measured, excised, and processed for immunoblotting. Extracts were resolved using electrophoresis and analyzed by immunoblotting. Vinculin and beta‐actin were used as loading controls. (G and H) PC9 cells (2.5 × 10^6^ cells per animal) were injected subcutaneously into the flanks of CD1‐nu/nu mice. Once tumors became palpable, mice (*n* = 7–9 per group) were randomized and treated for 3 weeks with the indicated antibodies, either Bis‐EM or the respective Fc‐mutated Bis‐EM, which were administered at 250 µg per injection, three times per week. Tumor volumes were measured twice weekly, and body weight was monitored weekly throughout the treatment period. Shaded areas indicate the treatment period. Both longitudinal tumor volume changes and the volumes on day 7 are shown. (I, J) NSG mice were implanted with fragments of the J6 patient‐derived xenograft (PDX) model harboring the EGFR exon 20 insertion mutation S768_V769insSVD. When tumors reached approximately 300 mm^3^, mice were randomized into the indicated treatment groups (7 mice per group). Mice were treated for 3 weeks (shaded areas) with Bis‐EM or with the Fc‐mutated Bis‐EM antibody, at 250 µg per injection, administered three times weekly. Tumor volumes and body weight were monitored twice weekly throughout the treatment period. Both longitudinal tumor volume changes and the volumes on day 17 are shown. Data are presented as mean ± SEM. Note: Original blots corresponding to panels (E, F) can be found in Figure S8A.

Unlike the exon 19 deletions and the L858R mutation in exon 21, which represent the majority of EGFR mutations in NSCLC, exon 20 insertions are relatively rare and confer a unique mechanism of kinase activation [55]. These structural attributes translate to lack of response to clinically approved EGFR inhibitors. Still, several compounds that selectively inhibit EGFR exon 20 insertions have been reported. One drug, poziotinib, showed activity in clinical trials but had dose‐limiting toxicities. Importantly, amivantamab, a bispecific EGFR/MET mAb, received approval in combination with chemotherapy for patients with EGFR exon 20 insertions [56]. Since all EGFR mutants retain a highly conserved extracellular domain, we examined whether Bis‐EM could also be effective against tumors harboring the difficult‐to‐treat exon 20 mutations. To this end, we used an exon 20 PDX model from The Jackson Laboratory (J000100672), established from a patient with the S768_V769insSVD mutation. Tumor fragments from this model were engrafted into mice and treated with different antibody regimens, including Bis‐EM, either alone or in combination with poziotinib, an exon 20 selective TKI. Because prolonged, high‐dose poziotinib has been linked to notable toxicities in patients, we administered a relatively low dose for a limited period of time to reduce the commonly observed delayed side effects [57]. As shown in Figure 7C,D and Figure S7B, poziotinib alone only partially delayed relapses of the exon 20 xenografts, and neither Bis‐EM nor the respective antibody combination (i.e., mAb745 plus cetuximab), when administered alone, blocked the aggressive growth of this model. However, both antibody treatments markedly potentiated the effect of poziotinib, even under low‐dose and short‐term conditions. Importantly, in similarity to the other xenograft experiments presented in this study, none of the TKI‐based combinations applied on the exon 20 PDX produced a consistent effect on animal body weight (Figure S7C–H).

To address the effects of Bis‐EM and poziotinib on receptor tyrosine kinases (RTKs), cell cycle and apoptosis in mice bearing exon 20 insertions, fragments of the human tumor were implanted in NSG mice and later treated as in Figure 7C, except that tumors were excised and processed for immunoblotting after only seven days of treatment. Thereafter, the relatively small tumors were excised and examined using immunoblotting (Figure 7E,F; each lane represents a single mouse). Unlike the relatively mild effects of Bis‐EM on RTK abundance and signaling proteins like ERK and AKT, the TKI accelerated degradation of EGFR, MERTK, and AKT. Similarly, poziotinib robustly inhibited cell cycle progression (as indicated by the disappearance of cyclin B1) and activated apoptosis, as reflected by caspase 3 cleavage and the induction of BIM, whose accumulation characterizes TKI‐sensitive tumors [58]. Overall, the results obtained with the exon 20 model indicated that despite relatively mild effects of Bis‐EM, when singly applied, on signaling and apoptosis, this EGFRxMERTK antibody can delay the emergence of resistance to poziotinib. Taken together, these findings suggest that next‐generation, less toxic exon 20–specific TKIs may synergize with Bis‐EM to provide durable clinical benefit, particularly when administered continuously rather than intermittently, as in the present study.

### 2.10 The Fc Region of the Bis‐EM is Essential for Inhibiting Tumor Growth In Vivo

A previous study indicated that cetuximab induces NK cell killing through Fc binding to CD16, which triggers ADCC [59]. To examine relevance to Bis‐EM, despite the use of immunocompromised mice and human‐derived models, we silenced the Fc region. To this end, asparagine 297 of Bis‐EM was mutated to alanine, and then we examined the effect of this well‐established way to nullify the ability of immunoglobulins to recruit effector immune cells. This modification is crucial for maintaining the Fc conformation required for binding to Fcγ receptors (FcγRs), including CD16/FcγRIIIa, CD32/FcγRII, and CD64/FcγRI. As shown in Figure 7G,H, when tested in CD‐1/nude mice (harboring no T cells but carrying macrophages, B and NK cells), the N297A mutant form of Bis‐EM partly lost the ability to inhibit the tumorigenic growth of PC9 cells. In contrast, when tested on a PDX model harboring an exon 20 mutant form, the wildtype and the mutant forms of Bis‐EM displayed indistinguishable, albeit weak abilities to inhibit the exon 20 PDX model in NSG mice. Critically, this animal strain lacks NK cells and has functionally impaired macrophages. Note that we observed no consistent effects of Bis‐EM and the Fc mutated version on animal body weights. In conclusion, silencing the Fc region of Bis‐EM may abrogate its anti‐tumor activity by impairing Fc‐mediated recruitment and activation of NK cells and other immune effector cells in the NSCLC models we examined.

In summary, our findings demonstrate that concurrent inhibition of either GAS6 or MERTK with EGFR can substantially delay the emergence of TKI resistance. Notably, we showed that the EGFRxMERTK bispecific antibody, Bis‐EM, is effective across diverse EGFR‐driven NSCLC models, including tumors harboring the clinically challenging exon 20 mutations. Although combinations of MERTK‐ and EGFR‐selective TKIs exhibited comparable activity in vitro, Bis‐EM showed superior efficacy across several in vivo models. Consistent with these findings, our data provide evidence that the Fc region of Bis‐EM, together with immune effector cells, contributes to its ability to delay the emergence of drug resistance in vivo.

## 3 Discussion

MERTK has previously been implicated in resistance to diverse anticancer therapies. For instance, this receptor activates pro‐survival signaling that diminishes the effectiveness of chemotherapy, and its inhibition sensitizes tumor cells to cytotoxic agents [60]. MERTK also contributes to an immunosuppressive tumor microenvironment that reduces the efficacy of radiotherapy [61]. Moreover, resistance to targeted kinase inhibitors—such as EGFR or AXL‐specific TKIs—can involve MERTK, which functions as a bypass node that reactivates downstream survival pathways [62]. One key to understanding the roles played by MERTK in drug resistance is the rather unique ability of this receptor to mediate efferocytosis, the process by which apoptotic cells are recognized and engulfed by macrophages and dendritic cells [63]. The ligands of TAM family receptors, GAS6 and Protein S, serve as bridging molecules by binding phosphatidylserine exposed on dying cells while simultaneously engaging MERTK and other TAM receptors on phagocytes, thereby driving apoptotic cell clearance. This mechanism might be relevant to our initial observation that made use of a clinical dataset: both GAS6 and MERTK—the most critical mediators of efferocytosis—were found to be upregulated in tumors of lung cancer patients shortly after initiation of TKI treatment. We therefore hypothesize that extensive drug‐induced apoptosis, followed by efferocytosis, activates MERTK as a bypass survival pathway. Hence, we assume, blocking either GAS6 or MERTK might delay the onset of resistance to osimertinib.

To test this survival model, we developed three antibodies: two mAbs—one targeting GAS6 and the other MERTK—and a bispecific antibody, Bis‐EM, directed against both MERTK and EGFR. Bispecific antibodies (BsAbs) like Bis‐EM offer several advantages over single‐target mAbs. By simultaneously engaging two antigens, they can enhance specificity and efficacy, improve safety, and simplify treatment regimens [64]. Critically, by blocking multiple pathways at once, BsAbs can counteract tumor escape mechanisms and prevent the development of resistance that often limits the efficacy of single target mAbs. This is especially relevant in the context of EGFR‐specific TKIs, where adaptive activation of bypass RTKs, including EGFR, HER2, HER3, AXL, MET, MERTK, or IGF1R, is a common event. Inhibiting individual bypass pathways has been shown to partially delay relapse [32, 36, 65, 66, 67, 68, 69]. A clinically validated example of this approach is amivantamab, a bispecific IgG1 antibody directed against EGFR and MET. Unlike TKIs, amivantamab not only drives receptor internalization and degradation but also enhances antibody‐dependent cellular cytotoxicity (ADCC) [70]. Similarly, our in vivo studies that made use of an Fc‐silent version of Bis‐EM implicated our bispecific antibody in ADCC. In addition, Bis‐EM promotes degradation of both EGFR and MERTK and, in our animal models, markedly delays the onset of resistance to TKIs, including an exon 20–selective inhibitor. Collectively, these attributes identify Bis‐EM as a compelling candidate for clinical translation. Especially important, the strategy we introduced may be relevant for patients with the difficult‐to‐treat EGFR exon 20 insertions. When identified at diagnosis, such patients may receive first‐line therapy with amivantamab plus chemotherapy. Upon disease progression, amivantamab monotherapy or TKIs are typically considered. However, using an exon 20 PDX model, we observed strong synergy between Bis‐EM and an exon 20–selective TKI, pointing to an alternative therapeutic strategy.

As expected, both anti‐MERTK and anti‐GAS6 antibodies delayed tumor relapse, underscoring the critical role of the GAS6–MERTK axis in TKI resistance. However, blocking GAS6 or MERTK alone was insufficient; maximal suppression required concurrent EGFR inhibition with both osimertinib and cetuximab. These findings support the ability of GAS6–MERTK signaling to provide an alternative survival route when EGFR is suppressed. Notably, although a MERTK kinase inhibitor was active in 3D cultures, its efficacy was markedly reduced in vivo. In contrast, the antibodies tested showed superior in vivo activity, likely reflecting their ability to engage immune effector cells that persist in the immunocompromised mice used here. Consistent with this possibility, osimertinib‐induced tumor regression has been associated with local accumulation of tumor‐associated macrophages [71]. Importantly, Bis‐EM showed in vivo activity comparable to its parental antibody combination despite 4‐fold and 10‐fold reductions in MERTK and EGFR affinity, respectively. This may reflect avidity‐driven engagement of both antigens on the same tumor cell: binding of one arm increases the effective local concentration of the second antigen, thereby reducing overall dissociation. Consistent with this concept, affinity‐attenuated bispecific antibodies have been shown to retain potent activity through dual‐antigen, avidity‐driven engagement [72].

In summary, we initially observed that upregulation of both GAS6 and its major receptor, MERTK, coincided with the onset of clinical resistance to osimertinib, suggesting that the GAS6–MERTK axis mediates drug escape. This hypothesis was supported by mAbs targeting GAS6 or MERTK, each of which partially delayed TKI resistance. We subsequently streamlined this approach by engineering a highly potent EGFRxMERTK bispecific antibody. Importantly, the observations we made herein using a previously described EGFRxAXL bispecific antibody [40] suggest that the GAS6‐AXL pathway might similarly mediate resistance to TKIs. Together, our findings reveal clinically relevant receptor crosstalk and establish the GAS6–MERTK axis as both a contributor to drug resistance and a potential therapeutic target. More broadly, these findings support a therapeutic strategy for patients with EGFR‐mutant lung cancer, including those harboring exon 20 mutations. By combining EGFR inhibition with Bis‐EM‐mediated dual targeting of EGFR and MERTK, this approach may provide a means of overcoming adaptive resistance and delaying its emergence, potentially extending the duration of treatment benefit beyond that achieved with TKI monotherapy alone.

## 4 Experimental Methods

### 4.1 Cell Culture

PC9 cells were purchased from Sigma (RRID: CVCL_B260) and both H1975 (RRID: CVCL_1511) and HEK293T ((RRID: CVCL_0063)) cells were from ATCC. Cells were grown with either RPMI‐1640 or DME medium supplemented with 10% fetal bovine serum (FBS) and Penicillin‐streptomycin at 37°C under 5% CO_2_. Osimertinib (Cat. No.: HY‐15772) and MRX‐2843 (Cat. No.: HY‐101549) were purchased from MedChemExpress. Recombinant cetuximab was expressed and produced in an Expi293 (Thermo Fisher Scientific Cat# A14527) Expression System. The anti‐GAS6 and anti‐MERTK antibodies were generated using the hybridoma technology.

### 4.2 Animal Studies and PDX Models

All studies were approved by the Weizmann Institute's Animal Care and Use Committee (IACUC), which serves as the Institutional Review Board (IRB). The following approval numbers are relevant to this study: 06120823‐3, 06090823‐2, 06150823‐3, 08821122‐2, 04180521‐3, 06560821‐1 and 08941121‐1. CD1 nude mice were used for cell line xenograft studies, whereas NSG mice were used in experiments that tested PDX models. PC9 and H1975 cells (2.5 × 10^6^ per animal) were subcutaneously injected into the right flanks of 5–6‐week‐old female CD1 nude mice. Once tumors became palpable, the mice were randomized into treatment groups and administered TKIs via daily oral gavage or mAbs, twice weekly intraperitoneally. The PDX models, TM00193 and J000100672, were obtained from The Jackson Laboratory. Tumor fragments were implanted subcutaneously into the lower backs of 8–10‐week‐old male or female NSG mice (RRID: IMSR_JAX:005557). The procedure was performed under anesthesia, and the surgical site was closed using clips. When tumors reached approximately 350 mm^3^, mice were randomized into experimental groups as specified. Tumor volume was monitored twice weekly and calculated using the formula: (T1 × T2 × (T1 + T2)/2) / 2, where T1 and T2 are the tumor's longest and shortest dimensions, respectively. Animals’ weights were determined once per week. Animals showing signs of illness were humanely sacrificed. The experimental endpoint was euthanasia by CO_2_ administration.

### 4.3 Generation of MERTK‐ and GAS6‐Overexpressing Cells

To generate PC9 cells overexpressing GAS6 or MERTK‐eGFP we made use of lentiviral particles. These cells were co‐transfected with second‐generation packaging plasmids pMD2.G (RRID: Addgene_12259) and psPAX2 (RRID: Addgene_12260) along with lentiviral expression vectors encoding either MERTK‐eGFP or GAS6. The resulting viral particles were harvested and used to transduce PC9 cells, which were then selected under puromycin to establish stably overexpressing cell lines.

### 4.4 CRISPR/Cas9‐Mediated Knockout of MERTK and GAS6

The CRISPR–Cas9 technology was used to knockout the MERTK and GAS6 genes. Target sites were selected adjacent to the Protospacer Adjacent Motif (PAM) sequence using the ENSEMBL database (Ensembl (RRID:SCR_002344)). A double‐stranded break was introduced at the selected locations to disrupt the respective gene transcripts.

### 4.5 Cell Viability and Proliferation Assays

Cells (1 × 10^3^ per well) were seeded in 96‐well plates. After 24 h, they were treated with the indicated drugs for 72 h. Following treatment, MTT solution (0.5 mg/mL) was added and incubated for 4 h to allow for formazan crystal formation. DMSO was then added to dissolve the crystals, and absorbance was measured at 570 nm using a microplate reader. Alternatively, cells were seeded in 96‐well plates and treated daily from Day 1 to Day 9, as indicated. On the designated days, cell proliferation was measured using crystal violet staining. Briefly, cells were fixed with ice‐cold methanol for 20 min, followed by staining with 1% crystal violet in methanol for 20 min at room temperature. After washing and drying, SDS (2%) was added to solubilize the dye, and absorbance was measured at 490 nm.

### 4.6 Colony Formation and Drug‐Tolerant Persister (DTP) Assays

For colony formation assays, cells (1000 per well) were seeded in 6‐well plates. For DTP assays, cells were seeded at full confluency in 6‐well plates. Treatments were applied the following day, with media refreshed every 3 days. After 7–10 days, cells were fixed with ice‐cold methanol for 20 min and stained with 0.5% crystal violet in methanol for another 20 min at room temperature. Colonies were scanned using a Photo Scanner (Epson Perfection 4870). Images from five non‐overlapping fields were captured using a light microscope (Olympus Corporation). Quantification was performed using ImageJ (ImageJ (RRID:SCR_003070)).

### 4.7 Cell Cycle Analyses Using FUCCI

Cell‐cycle progression was monitored using the ubiquitination‐based fluorescent cell cycle indicator (FUCCI) system, which enables real‐time visualization of interphase and cell‐cycle distribution [52]. Lentiviral particles were produced in HEK293T cells by co‐transfecting Fucci (CA)2/pCSII‐EF (RIKEN BRC, Tsukuba, Japan) together with the packaging plasmids psPAX2 (Addgene #12260) and pMD2.G (Addgene #12259) using FuGene HD reagent (Promega). PC9 cells were subsequently infected with the viral supernatant, and stable FUCCI‐expressing cells were sorted using a cell sorter. Cells were then treated for three days with the indicated agents, either alone or in combination, including osimertinib (10 nm), MRX‐2843 (300 nm), cetuximab (10 µg/mL), mAb745 (10 µg/mL) and Bis‐EM (20 µg/mL). Cellular images were captured daily from at least nine distinct fields of view using a CV8000 high‐content imaging system (Yokogawa, Tokyo, Japan). Nuclei were identified based on FUCCI fluorescence signals, and circularity thresholds were applied to exclude fluorescent debris. The intensity of green and red fluorescence signals was quantified to assign cells to G1, S, or G2/M phases. Image processing was performed using Fiji, followed by segmentation and quantification with CellProfiler. Data visualization and analysis used R Studio.

### 4.8 Spheroid Cytotoxicity and Apoptosis Assays

For the experiment shown in Figure 5C and Figure S5B, cells (1 × 10^3^) were seeded in a BME pre‐coated 96‐well plate and embedded in 5% BME medium containing drugs. The experiments were stopped after 7 (PC9 cells) or 14 days (PC9‐AZDR cells). Photos were captured using an OpTech IB4 microscope, in brightfield (20X magnification). Percentages of covered area were assessed using ImageJ. For other experiments, PC9 cells were seeded at 3 × 10^3^ cells per well in 96‐well ultra‐low‐attachment plates and cultured to allow spheroid formation. Next, spheroids were treated with monoclonal antibodies (10 µg/mL) or with the bispecific antibody Bis‐EM (20 µg/mL). They were monitored for apoptosis using the IncuCyte caspase‐3/7 assay. The caspase‐3/7 reagent is a non‐fluorescent DEVD‐based substrate that, upon cleavage by activated caspase‐3/7, releases a fluorescent DNA‐binding molecule, resulting in nuclear fluorescence in apoptotic cells. For co‐cultures containing peripheral blood mononuclear cells (PBMCs), image analysis was configured to restrict quantification of caspase‐3/7 fluorescence to the spheroid‐associated region rather than measuring total green fluorescence across the entire well. Specifically, the IncuCyte metric “Largest Brightfield Object Green Mean Intensity (GCU)” was used, whereby spheroids were identified as the largest brightfield objects and the mean green fluorescence intensity was quantified within this region. Consequently, caspase‐3/7‐positive PBMCs located outside the segmented spheroid region were excluded from the analysis.

### 4.9 Perforin Quantification Using ELISA

Perforin secretion was quantified in culture supernatants using a Human Perforin Sandwich ELISA Kit (Invitrogen, Catalog #BMS2306), following the manufacturer's instructions. Briefly, samples, standards, and controls were added to microplate wells pre‐coated with a perforin‐specific capture antibody. After incubation, a detector antibody recognizing a different perforin epitope was added to form an antibody–perforin–antibody sandwich complex. Following washing steps, a substrate solution was applied, and the resulting colorimetric signal—proportional to perforin concentration—was measured and compared with a standard curve to determine perforin levels in each sample.

### 4.10 RNA Isolation and Real‐Time PCR

Total RNA was extracted using the RNeasy Mini Kit (QIAGEN), following the manufacturer's instructions. cDNA synthesis was performed using the qScript cDNA Synthesis Kit (Quantabio). Real‐time PCR was conducted using SYBR Green Master Mix (Applied Biosystems) and gene‐specific primers (see below). Reactions were run on a Real‐Time PCR system (Applied Biosystems). GAPDH served as an internal control for normalization using the ΔCt method. The following primers were used for GAS6: CCGGGGACTTGTTCCAACC (forward) and CTGCACGAGGTCCTTCTCAT (reverse). For Gpadh we used ACAACTTTGGTATCGTGGAAGG (forward) and GCCATCACGCCACAGTTTC (reverse).

### 4.11 Cell Migration Assays

Three different PC9 sublines were used to assess cell migration using a transwell assay. Briefly, 5 × 10^4^ cells (5 × 10^4^) were seeded in the upper chamber of Transwell inserts in serum‐free medium. The lower chamber was filled with RPMI medium supplemented with 10% serum to serve as a chemoattractant. Cells were allowed to migrate for 18 h under standard incubation conditions. Following incubation, non‐migrated cells on the upper surface of the membrane were gently removed. Cells that had migrated to the lower surface of the Transwell membrane were fixed with ice‐cold methanol and then stained with 0.5% crystal violet dissolved in methanol. Images of the stained membranes were captured using a bright‐field microscope, and the number of migrated cells was quantified using ImageJ software.

### 4.12 Immunofluorescence Analyses

Cells (1 × 10^4^ per well) were seeded on glass coverslips in a 24‐well plate. After three days, cells were fixed with paraformaldehyde (4%; PFA) at room temperature for 15 min, followed by two washes with saline. Cells were then blocked and permeabilized for 60 min at room temperature using a solution containing 1% BSA and 0.3% Triton X‐100. After blocking, cells were incubated overnight at 4°C with an anti‐Ki67 primary antibody (Cell Signaling Technology Cat#9449; RRID: AB_2797703). The following day, cells were washed with saline containing 0.05% Tween‐20 and incubated with an Alexa Fluor 488‐conjugated secondary antibody. DAPI and Rhodamine B‐conjugated phalloidin were used for nuclear and actin cytoskeleton staining, respectively. Images were captured using a Zeiss spinning disk confocal microscope and processed with Zeiss ZEN 3.1 software. ImageJ was used for signal quantification.

### 4.13 Immunostaining and Quantification of Internalized Receptors

PC9 cells (5 × 10^4^) were treated with either mAb745 (10 µg/mL), cetuximab (10 µg/mL), or the bispecific antibody Bis‐EM (20 µg/mL). Treated cells were fixed with PFA (4%) for 20 min at the following time‐points: 0h, 2h, 4h, and 8h. Fixed cells were subsequently permeabilized with 0,01% Triton‐X100 for 5 min to enable intracellular labeling. Cells were blocked with a solution of PBS containing BSA (1%). Subsequently, immunofluorescence staining was performed using antibodies directed against EGFR (ab289889, 1:500) and MERTK (CST#4319S, 1:100). Cells were incubated in primary antibody for 60 min, washed 3 times in saline, and secondary antibodies specific to the mouse section of the anti‐EGFR antibody and the rabbit section of MERTK were used. Coverslips were then mounted using the ProLong Gold Antifade Mountant (P36934). Images were taken using a Zeiss inverted spinning disk confocal equipped with a 60X objective. Several Z‐Stack (1 µm Z‐axis) were taken for analysis. To quantify the internalized receptors, the middle section of the cells was selected from the Z‐Stack, and for each channel the average signal in the cytosol of the cells was calculated. The resulting signal intensity was used as a thresholding value to remove the background cytosolic noise. The remaining spots were counted using the function “analyze particles”. To quantify and compare the different treatments, the average value of time zero of each treatment was used to normalize each sample.

### 4.14 Immunoprecipitation and Immunoblotting Analyses

Cells were washed with saline and extracted on ice using a buffer containing 50 mm HEPES (pH 7.5), 10% glycerol, 150 mm NaCl, 1% Triton X‐100, 1 mm EDTA, 1 mm EGTA, 10 mm NaF, 25 mm β‐glycerophosphate, 0.1 mm sodium orthovanadate and a protease inhibitor cocktail. Lysates were centrifuged at 14 000 × g for 20 min at 4°C, and the supernatants were collected for immunoprecipitation or immunoblotting. For immunoprecipitation, protein G beads were incubated overnight at 4°C with the indicated primary antibodies and cell lysates on a mechanical rotor. Beads were washed with ice‐cold HNTG buffer (20 mm HEPES pH 7.5, 150 mm NaCl, 0.1% Triton X‐100, 10% glycerol). Bound proteins were eluted by boiling in Laemmli buffer (Bio‐Rad) and resolved using gel electrophoresis. This was followed by transfer to nitrocellulose membranes. Membranes were blocked in 3% albumin, incubated overnight at 4°C with primary antibodies, and then with HRP‐conjugated secondary antibodies (1 h at room temperature). Blots were visualized using Clarity Western ECL Substrate (Bio‐Rad) and imaged on a ChemiDoc Imaging System (Bio‐Rad) and Image Lab v6.0.1 (RRID:SCR_014210). The table below lists the antibodies we used.

**Table:** 

Target	Catalog No.	Vendor	RRID
GAS6	67202	Cell Signaling Technology (CST)	AB_2799720
MERTK	4319S	Cell Signaling Technology (CST)	AB_10614528
AXL	8661	Cell Signaling Technology (CST)	AB_11217435
TYRO3	5585S	Cell Signaling Technology (CST)	AB_10706782
EGFR	4267	Cell Signaling Technology (CST)	AB_2246311
pEGFR (Tyr1068)	2234S	Cell Signaling Technology (CST)	AB_331701
HER2	2165S	Cell Signaling Technology (CST)	AB_10692490
HER3	12708	Cell Signaling Technology (CST)	AB_2721919
MET	8198S	Cell Signaling Technology (CST)	AB_10858224
AKT	2938	Cell Signaling Technology (CST)	AB_2315049
pAKT1 (Ser473)	4060	Cell Signaling Technology (CST)	AB_329825
ERK1/2	4695S	Cell Signaling Technology (CST)	AB_390779
pERK1/2 (Thr202/Tyr204)	9101S	Cell Signaling Technology (CST)	AB_331646
PARP1	9542S	Cell Signaling Technology (CST)	AB_2160739
Caspase‐3	9662S	Cell Signaling Technology (CST)	AB_331439
Cleaved Caspase‐3	9661S	Cell Signaling Technology (CST)	AB_2341188
Bak	12105	Cell Signaling Technology (CST)	AB_2716685
Bcl‐xL	sc‐634	Santa Cruz Biotechnology	AB_630917
BIM	2933S	Cell Signaling Technology (CST)	AB_1030947
Cyclin B1	12231S	Cell Signaling Technology (CST)	AB_2783553
γH2AX (Ser139)	9718	Cell Signaling Technology (CST)	AB_2118009
GAPDH	MAB374	Millipore	AB_2107445
α‐Tubulin	3873	Cell Signaling Technology (CST)	AB_1904178
Vinculin	V9264	Sigma‐Aldrich	AB_10603627
β‐Actin	3700	Cell Signaling Technology (CST)	AB_2242334

### 4.15 Sandwich ELISA

A custom sandwich ELISA was performed to assess the simultaneous binding of Bis‐EM to MERTK and EGFR. ELISA plates were coated overnight with the MERTK antigen, blocked with 3% BSA, followed by incubation with Bis‐EM for 60 min at room temperature. Next, the plate was incubated with an AVI‐tagged soluble form of EGFR (SinoBiological Cat: 10001‐H27H‐B). Detection was carried out using Streptavidin‐HRP, and absorbance was measured at 415 nm using a microplate reader.

### 4.16 NanoBRET Assays

HEK293T cells were seeded at a density of 500 000 cells per well into 6 well plates (Corning) and left to grow at 37°C/5% CO2 overnight. The next day, cells were transiently transfected with 500ug/well of EGFR and MERTK constructs, N‐terminally tagged with NLuc (energy donor) or HaloTag (energy acceptor) to give a total of 1000ug cDNA per well using FuGENE HD (Promega Corporation) at a 3:1 DNA: reagent ratio in OptiMEM (Gibco) following the manufacturer's instructions. The next day, cells were seeded onto white flat bottom 96 well microplates (Grenier Bio‐One 655098) at a density of 40 000 cells per well. The next day, the medium was replaced with prewarmed assay buffer (HEPES‐buffered saline solution supplemented with 0.1% protease‐free bovine serum albumin (Sigma); termed HBS/0.1% BSA). Cells were then incubated with 0.2 µm membrane impermeable HaloTag AlexaFluor488 (Promega Corporation) substrate for 30 min at 37°C.  Cells were then washed three times with HBS/0.15 BSA before being treated with the indicated growth factors. After incubation for 60 min at 37°C, the NanoGlo substrate furimazine (1:400 dilution) was added, and cells were further incubated for 5 min. Simultaneous fluorescence and luminescence emissions were then detected using a PHERAstar FSX plate reader (BMG LabTech), fitted with a bandpass filter to detect donor emission (NLuc emission) at 475 ± 30 nm and a bandpass filter to detect acceptor emission (HaloTag AlexaFluor488) at 535 ± 30 nm. BRET ratios were calculated by dividing fluorescence by luminescence emissions and baseline corrected to NLuc donor‐only conditions.

### 4.17 Proximity Ligation Assays (PLA)

Cells were plated on glass coverslips, fixed, permeabilized, and blocked using standard protocols. Primary antibodies were applied as indicated. The DuoLink in Situ PLA Detection Kit (Sigma–Aldrich) was used according to the manufacturer's instructions. This included incubation with PLA probes, ligation, signal amplification, and detection. Images were acquired using a Zeiss LSM800 confocal microscope.

### 4.18 Surface Plasmon Resonance (SPR) Measurements

SPR was performed using the Biacore T200 system to determine the binding affinities of the bispecific antibody Bis‐EM and its parental antibodies—anti‐MERTK (mAb745) and Cetuximab (CTX). Sensor Chip CM5 (Cytiva; Cat# BR100530) was activated using a freshly prepared solution of N‐hydroxysuccinimide (50 mm) and 1‐ethyl‐3‐(3‐dimethylaminopropyl; 195 mm) carbodiimide to introduce reactive amine groups. MERTK and EGFR proteins were immobilized onto designated flow cells via covalent attachment (approximately 300 RU). Unreacted groups were blocked using ethanolamine hydrochloride (1 m, pH 8.0). Various concentrations of the antibodies were injected, and sensograms were recorded. After each cycle, the chip's surface was regenerated using 2 mm NaOH. Data were analyzed using a steady‐state affinity model of the Biacore T200 Evaluation Software.

### 4.19 Drug Synergy Analysis

Cells (1 × 10^3^ per well) were seeded in 96‐well plates and treated for three days with TKIs, either as monotherapies or in combinations, at varying concentrations. Thereafter, cells were fixed with ice‐cold methanol for 20 min and stained with 1% crystal violet in methanol for another 20 min at room temperature. After staining, 2% SDS was used to solubilize the dye, and absorbance was measured at 595 nm using a microplate reader. Cell viability was calculated relative to untreated controls. Drug synergy scores (osimertinib and MRX‐2843) were quantified online using the Bliss model via SynergyFinder (https://github.com/IanevskiAleksandr/SynergyFinder#readme) (RRID:SCR_026127).

### 4.20 Generation of Monoclonal Antibodies

Balb/c mice were immunized with the extracellular domain of MERTK (SinoBiological, Cat: 10298‐H03H; 25 µg per injection). Blood samples were collected after the third and fifth immunizations to assess serum antibody titers. After seven total immunizations, including a final booster, mice were sacrificed, and splenocytes were harvested. These were fused with NSO myeloma cells to generate hybridomas, which were screened using ELISA and validated using immunoprecipitation. Positive clones were subcloned and selected. Anti‐GAS6 antibodies were generated using a similar hybridoma approach. Monoclonal antibodies were purified from hybridoma supernatants using protein G affinity chromatography.

### 4.21 Design, Expression, and Purification of Recombinant Antibodies

The heavy and light chain sequences of cetuximab were retrieved from public databases, while those of mAb745 were obtained from our hybridoma clones. DNA sequences encoding the variable regions were cloned into mammalian expression vectors containing human IgG1 and kappa light chain constant regions. For the design of the bispecific antibody, the EGFR‐binding arm was formatted as a CrossMab (CH1–CL swap) to ensure correct heavy–light chain pairing. Heterodimerization of heavy chains was achieved through the introduction of mutations in the CH3 domains: Y349C/T366S/L368A/Y407V (EGFR arm) and S354C/T366W (MERTK arm). The Fc‐silent mutant of Bis‐EM was generated by using site‐directed mutagenesis on the wild‐type IgG1 backbone. Specific primers were used to substitute the asparagine residue at position 297 within the CH2 domain with an alanine (N297A), thereby disrupting the Fc asparagine‐linked glycosylation site. The resulting plasmid was sequenced and verified for the mutation. All antibodies were produced in Expi293 cells (ThermoFisher) using transient transfection and purified from the culture supernatant using protein G chromatography.

### 4.22 Amino Acid Sequences of the Four Chains of Bis‐EM

Note: the variable domain sequences are shown in bold type.

(i) Anti‐MERTK (mAb745), heavy chain (‘knob’ mutations are underlined)

MGWSCIILFLVATATGVHS**DVQIQESGPGLVKSSQSLSLTCTVTGYSITNDYAWNWIRQFPGNKLEWMGYISYNGNTFYNPSLKSRISITRDTSKNQFFLQLSSVTTEDTATYYCGRLLPVSYAMDYWGQGTSVTVSS**ASTKGPSVFPLAPSSKSTSGGTAALGCLVKDYFPEPVTVSWNSGALTSGVHTFPAVLQSSGLYSLSSVVTVPSSSLGTQTYICNVNHKPSNTKVDKRVEPKSCDKTHTCPPCPAPELLGGPSVFLFPPKPKDTLMISRTPEVTCVVVDVSHEDPEVKFNWYVDGVEVHNAKTKPREEQYNSTYRVVSVLTVLHQDWLNGKEYKCKVSNKALPAPIEKTISKAKGQPREPQVYTLPPCREEMTKNQVSLWCLVKGFYPSDIAVEWESNGQPENNYKTTPPVLDSDGSFFLYSKLTVDKSRWQQGNVFSCSVMHEALHNHYTQKSLSLSPGK

(ii) Anti‐MERTK (mAb745) light chain

MGWSCIILFLVATATGVHS**DVVMTQTPLSLPVSLGDQASISCRSSQSLVHSNGNTYLHWYLQKPGQSPKLLIYKVSNRFSGVPDRFSGSGSGTDFTLKISRVEAEDLGVYFCYQSTHVPWTFGGGTKLEIK**RTVAAPSVFIFPPSDEQLKSGTASVVCLLNNFYPREAKVQWKVDNALQSGNSQESVTEQDSKDSTYSLSSTLTLSKADYEKHKVYACEVTHQGLSSPVTKSFNRGEC

(iii) Anti‐EGFR (cetuximab), heavy chain (‘hole’ mutations shown are underlined)

MGWSCIILFLVATATGVHS**QVQLKQSGPGLVQPSQSLSITCTVSGFSLTNYGVHWVRQSPGKGLEWLGVIWSGGNTDYNTPFTSRLSINKDNSKSQVFFKMNSLQSNDTAIYYCARALTYYDYEFAYWGQGTLVTVSS**ASVAAPSVFIFPPSDEQLKSGTASVVCLLNNFYPREAKVQWKVDNALQSGNSQESVTEQDSKDSTYSLSSTLTLSKADYEKHKVYACEVTHQGLSSPVTKSFNRGECDRSEPKSCDKTHTCPPCPAPELLGGPSVFLFPPKPKDTLMISRTPEVTCVVVDVSHEDPEVKFNWYVDGVEVHNAKTKPREEQYNSTYRVVSVLTVLHQDWLNGKEYKCKVSNKALPAPIEKTISKAKGQPREPQVCTLPPSREEMTKNQVSLSCAVKGFYPSDIAVEWESNGQPENNYKTTPPVLDSDGSFFLVSKLTVDKSRWQQGNVFSCSVMHEALHNHYTQKSLSLSPGKSAWSHPQFEK

(iv) Anti‐EGFR (cetuximab), light chain, CrossMab

MGWSCIILFLVATATGVHS**DILLTQSPVILSVSPGERVSFSCRASQSIGTNIHWYQQRTNGSPRLLIKYASESISGIPSRFSGSGSGTDFTLSINSVESEDIADYYCQQNNNWPTTFGAGTKLELK**SSASTKGPSVFPLAPSSKSTSGGTAALGCLVKDYFPEPVTVSWNSGALTSGVHTFPAVLQSSGLYSLSSVVTVPSSSLGTQTYICNVNHKPSNTKVDKKVEPKSC

### 4.23 Anti‐EGFRxAXL Bispecific Antibody

The structure and purification of the anti‐EGFR/AXL bispecific antibody, Bis‐EA, were previously reported [40].

### 4.24 Bioinformatics Analyses

The Kaplan–Meier Plotter tool (https://kmplot.com/analysis/) was used to evaluate the prognostic significance of MERTK expression in cancer patients. To perform the analysis, patient cohorts were selected based on available gene expression and survival data. The hazard ratio (HR) and corresponding p‐values were automatically calculated by the tool and displayed on the survival plots. The data sources integrated into the Kaplan–Meier Plotter included several publicly available datasets from GEO and TCGA, covering lung adenocarcinoma patients from the following datasets (patient number) GSE102287 (*n* = 66), GSE14814 (*n* = 90), GSE157011 (*n* = 235), GSE19188 (*n* = 156), GSE29013 (*n* = 55), GSE30219 (*n* = 307), GSE31210 (*n* = 246), GSE3141 (*n* = 111), GSE31908 (*n* = 40), GSE37745 (*n* = 196), GSE43580 (*n* = 150), GSE4573 (*n* = 130), GSE50081 (*n* = 181), GSE68465 (*n* = 462), GSE77803 (*n* = 156), GSE8894 (*n* = 138) and TCGA (*n* = 133). Single‐cell RNA sequencing data were taken from a clinical trial, NCT03433469. Patients were treated with osimertinib, and the cohort was segregated into Treatment Naïve (TN), Residual Disease (RD), and Progressive Disease (PD). EGFR+ NSCLC patient samples were analyzed for differentially expressed genes (DEGs). Expression was compared between cells in the different time points using Wilcoxon's test within the Seurat package, using R v4.2.1 (RRID:SCR_001905)).

### 4.25 Statistical Analyses

GraphPad Prism (version 10.6.0; GraphPad Prism (RRID:SCR_002798)) was used to perform statistical analyses. Sample numbers and other information (mean ± SEM, number of replicates, and specific statistical tests) are indicated in the respective figure legends. We employed one‐way ANOVA followed by Dunnett's multiple comparison test. The ImageJ software packages were used to perform data analysis. Statistical significance was denoted as follows: *p* < 0.05 (^*^), *p* < 0.01 (^**^), *p* < 0.001 (^***^), and *p* < 0.0001 (^****^). Differences were considered statistically significant when *p* < 0.05. Unless otherwise indicated, all in vitro experiments were repeated twice.

## Author Contributions

Conceptualization, S.G. and Y.Y.; Methodology, S.G., B.R.S., A.S.N., N.G., M.L., R.C., A.G. M.V.D., D.R. K., L.R.P., M.Z., R.O., I.S.M., E.F.W., L.E.K., S.P., D.R., Y.H., E.H.Y., Y.A. and R.D.; Formal Analysis, S.G., B.R.S., M.A.A, O.R., A.S‐N., N.G., M.L. and L.E.K.; Investigation, S.G., B.R.S.,  A.S.N., N.G., M.L., L.E.K.; Resources, S.P., S.J.H., M.L., Y.T. and Y.Y.; Writing – Original Draft, S.G. and Y.Y.;  Writing – Review, all authors; Supervision, E.R., S.J.H., M.L., R.D. and Y.Y.;  Project Administration, Y.Y. Funding acquisition, S.J.H., E.R., A.P. and Y.Y.

## Funding

Our studies were supported by the Israel Science Foundation (grants received by AP; 3444/25 and YY; 1931/20), the European Research Council (ERC; 740469), the Israel Cancer Research Fund (ICRF; 18‐304), the Dr. Miriam and Sheldon G. Adelson Medical Research Foundation (grant number G0202206) and the Medical Research Council, UK (grant number MR/w016176/1).

## Conflicts of Interest

The authors declare no conflicts of interest.

## Supporting information




**Supporting File**: advs78290‐sup‐0001‐SuppMat.docx.

## Data Availability

The data that support the findings of this study are available on request from the corresponding author. The data are not publicly available due to privacy or ethical restrictions.
