# Supplementary material for: Fc‐Dependent Targeting of the GAS6–MERTK Axis With Monoclonal and Bispecific Antibodies Overcomes Drug Resistance Across EGFR Mutations in Exons 19, 20, and 21
Source: Adv Sci (Weinh). 2026 Oct 14:e78290. Online ahead of print. doi: 10.1002/advs.78290 (PMC13669479; doi:10.1002/advs.78290)
Supplement: Supplementary file 1 [file ADVS-9999-e78290-s001.docx]

**Supplementary Figures and Legends:**

**Fc-dependent targeting of the GAS6–MERTK axis with monoclonal and bispecific antibodies overcomes drug resistance across EGFR mutations in exons 19, 20, and 21**

### Suvendu Giri^1^, Boobash Raj Selvadurai^1^, Nitin Gupta^1^, Arturo Simoni-Nieves^1^, Alessandro Genna^1^, Moshit Lindzen^1^, Rishita Chatterjee^1^, Marieke Van Daele ^1^, Deepthi Ramesh Kumar^1^, Mirie Zerbib^2^, Roni Oren^2^, Isabelle Sophie Meijer^1^, Elisabeth Franziska Wippich^1,3^, Yahel Avraham^4^, Rony Dahan^4^, Yuya Haga^5^, Laura E. Kilpatrick^6,7^, Simon Platt^6,8^, Stephen J. Hill^6,8^, Lipika R. Pal^9^, Eytan Ruppin^9^, Yasuo Tsutsumi^5,10,11,12,13^, Eliane Hadas Yardeni^14^, Muhammad Abu Ahmad^15^, Olga Radinsky^15^, Donatella Romaniello^16,17^, Mattia Lauriola^16,17^, Angel Porgador^15^ and Yosef Yarden^1,*^

Departments of ^1^Immunology and Regenerative Biology, ^2^Veterinary Resources, ^3^Molecular Cell Biology and ^4^System Immunology, Weizmann Institute of Science, Rehovot, Israel, ^5^Graduate School of Pharmaceutical Sciences, The University of Osaka, Osaka, Japan. ^6^Centre of Membrane Proteins and Receptors (COMPARE), University of Birmingham and University of Nottingham, Midlands, UK, ^7^Division of Bimolecular Science and Medicinal Chemistry, School of Pharmacy, Biodiscovery Institute, University of Nottingham, Nottingham, UK, ^8^Division of Physiology, Pharmacology and Neuroscience, School of Life Sciences, University of Nottingham, UK, ^9^Cancer Data Science Laboratory, Center for Cancer Research, National Cancer Institute, Bethesda, MD, USA, ^10^Graduate School of Medicine, ^11^Institute for Open and Transdisciplinary Research Initiatives, Suita, ^12^Global Center for Medical Engineering and Informatics, ^13^R3 Institute for Newly-Emerging Science Design, The University of Osaka, Osaka, Japan, ^14^Department of Life Science Core Facility, Weizmann Institute of Science, Rehovot, Israel, ^15^The Shraga Segal Department of Microbiology, Immunology and Genetics, Faculty of Health Sciences, Ben-Gurion University of the Negev, Beer-Sheva, Israel, ^16^Department of Medical and Surgical Sciences (DIMEC) and ^17^IRCCS Azienda Ospedaliero-Universitaria di Bologna, University of Bologna, Bologna, Italy

^*^To whom correspondence should be addressed: Yosef Yarden, Department of Immunology and Regenerative Biology, Weizmann Institute of Science, Rehovot 76100, Israel. Tel: 972-8-934 3974; E-mail: [yosef.yarden@weizmann.ac.il](mailto:yosef.yarden@weizmann.ac.il)

**Keywords:** Bispecific antibody, drug resistance, EGFR, GAS6, MERTK, osimertinib

**Running Title:** Targeting MERTK in EGFR mutated lung cancer

**Figure S1: Dataset analysis, characterization of GAS6-manipulated cells and anti-GAS6 antibodies.** (**A**) Data taken from NCT03433469 (EGFR-positive NSCLC patients) were analyzed for differentially expressed genes (DEGs). Patients were treated with osimertinib and segregated into Treatment Naïve (TN), Residual Disease (RD), and Progressive Disease (PD). The volcano plot presents DEGs that differ between TN (6 patients) and RD (4 patients). MERTK, AXL, GAS6, and EGFR are highlighted in red, and the average fold changes (log2FC), along with the *p*-values, are presented in a table. (**B** and **C**) mRNA and proteins were isolated from the indicated genetically modified PC9 cells, and the expression of GAS6 was assayed using RT-PCR (B) and immunoblotting (C). GAPDH served as a gel loading control. (**D**) Anti-GAS6 mAbs derived from two hybridoma clones (clones 3 and 197) were tested at different concentrations (1, 5, and 25 µg/ml) in an immunoprecipitation assay. HEK293 cells were transfected with a GAS6 expression plasmid and culture supernatants were collected 48 hours post-transfection. The supernatant was subjected to immunoprecipitation using the specified antibodies and the resulting samples were analyzed by immunoblotting (IB) that used the indicated antibodies. (**E**) The binding affinity of the anti-GAS6 mAb197 was evaluated using Surface Plasmon Resonance (SPR) assays and the Biacore™ T200 system. Human recombinant GAS6 was utilized in this binding assay. The sensor chip was activated and bound to the recombinant GAS6 protein. Next, different concentrations of the anti-GAS6 mAb197 were passed through the specific channels. Sensograms were generated and the data analyzed using a steady-state affinity model. (**F**) Average animal body weights were calculated, and shown. See Figure 1E for experimental details. All experiments were repeated at least twice. Note: Original blots corresponding to panels C and D can be found in Fig, S8B.

**Figure S2:** **MERTK is needed for PC9 cell proliferation**. (**A**) Wild-type, MERTK knockout (MKO) and MERTK overexpressing (MOX) PC9 cells were extracted and the lysates were prepared for western blot analysis, as indicated, to test MERTK’s expression level and adaptive effects on the abundance of AXL. Vinculin was used as a gel loading control. (**B**) Cells (2x10^4^/per well) were seeded on coverslips with 1% serum and cultured for 3 days. Next, they were fixed and stained overnight with an anti-Ki67 antibody (red). Nuclear DNA and actin filaments were respectively labeled with DAPI (blue) and phalloidin (green). Images were taken using a spinning disk confocal microscope and analyzed using ImageJ. The fractions of Ki67-positive cells, out of all DAPI-positive cells, were determined. The experiment was repeated twice. Scale bar, 4 µm. *, p<0.05; ***, p<0.001. (**C** and **D**) Parental PC9 cells or cells with CRISPR-mediated knockout of MERTK (MKO; panel C) or AXL (AKO; panel D) were treated with either erlotinib (50 nM) or osimertinib (10 nM) for 48 hours. Whole-cell lysates were subsequently prepared and analyzed using immunoblotting to assess the expression of bypass signaling receptors. In addition, the activation status of the downstream MAPK and AKT signaling pathways was evaluated. Vinculin and α-tubulin were used to ensure equal gel loading. (**E**) PC9 cells were treated for 72 hours with tyrosine kinase inhibitors. Cell viability was subsequently assessed using a colorimetric test used to estimate the number of metabolically active cells. Cells were cultured in 10% serum and treated with osimertinib (10 nM), MRX-2843 (300 nM) or bemcentinib (100 nM). Statistical significance was denoted as follows: p<0.05 (*), p<0.01 (**), p<0.001 (***), and p<0.0001 (****). All experiments were repeated at least twice. Note: Original blots corresponding to panels A, C and D can be found in Fig, S8B.

**Figure S3: Selection of an anti-MERTK mAb, BRET and xenograft studies**. (**A**) Human recombinant MERTK’s extracellular domain was injected into two mice at three-week intervals, for a total of six doses (25 µg/injection). Blood samples were collected in weeks 7 and 12, and the corresponding serum was used in an immunoprecipitation assay. PC9 cell lysate was used as the antigen source in this assay. As a positive control, we used a different anti-MERTK antibody. Pre-immunization serum was used as a negative control. The immunoblot shows the levels of anti-MERTK antibodies present in the serum in week 7. (**B**) Immunized animal number 2 was sacrificed in week 16, and the spleen was harvested for hybridoma generation. Approximately 1,000 hybridoma clones were established and initially screened for antibody titer using immunoprecipitation and PC9 cell lysates. A commercially available anti-MERTK antibody served as a positive control in this assay. (**C**) CD1-nu/nu mice bearing PC9 xenografts were randomized in groups of 5–7 animals and treated for 4 weeks with antibodies (200 µg per injection; intraperitoneally, twice weekly) or daily with osimertinib (5 mg/kg, oral gavage). Tumor volumes were measured twice weekly, and body weight was measured once weekly. The grey-shaded area marks the treatment period. Changes in tumor volume (mm³) from treatment onset are shown, with the number of surviving mice indicated in brackets (see Fig. 3A). (**D**) Shown is a scheme outlining the BRET assay. Note the donor (NanoLuc) and acceptor (HaloTag) we utlized. (**E**) HEK293 cells were transfected with NLuc-tagged and Halo-tagged EGFR and MERTK constructs (respectively). The following day, cells were treated with the indicated ligands and incubated for 60 minutes at 37°C. The NanoLuc substrate was then added, followed by a 15-minutes long incubation in the dark. Luminescence was measured using a PHERAstar FSX plate reader, a 475 ± 15 nm bandpass filter (donor) and 665/535 ± 15 nm bandpass filter (acceptor). Raw BRET ratios were calculated by subtracting fluorescence emissions from luminescence emissions as the 665/475 nm signal and baseline-corrected using the 'no acceptor' control. *, p<0.05; ns, non-significant. Note: Original blots corresponding to panels A and B can be found in Fig, S8B.

**Figure S4: Demonstration that Bis-EM can induce activation of caspase3/7 and enhance degradation of both MERTK and EGFR.** (**A**-**D**) The binding affinities of the indicated antibodies towards EGFR and MERTK were assessed by analyzing the association and dissociation kinetics of antigen-antibody complexes using surface plasmon resonance (SPR; Biacore T200 system). A recombinant MERTK protein was covalently immobilized on a sensor chip and various concentrations of anti-MERTK antibody (mAb745) were flown over the surface for 2 minutes, prior to a 10-minute dissociation phase. Bis-EM was tested on surfaces coated with both MERTK and EGFR in separate lanes, and the obtained signals were corrected by subtracting reference signals from empty lanes. The dissociation constant (Kd) was calculated as the ratio of the dissociation rate constant (koff) to the association rate constant (kon), and the values are summarized in Figure 4A. (**E**) Ninety-six-well plates were coated overnight with a soluble ectodomain of MERTK. The following day, the plates were blocked with albumin-containing saline, and thereafter they were incubated with the indicated antibodies (20 mg/mL), including C225 (a chimeric precursor of cetuximab), HM745 (mAb745 from hybridoma) and a recombinant form of mAb745 (human Fc). After washing, biotinylated EGFR’s ectodomain was added, followed by avidin–horseradish peroxidase. The signal was measured using a microplate reader. Note that both native and engineered (recombinant) versions of cetuximab (CTX) and mAb745 were used. (**F**) PC9 cell lysates were subjected to immunoprecipitation assays that used Bis-EM, anti-EGFR and anti-MERTK antibodies. The immunoblots were probed with the indicated antibodies. Recombinant forms of mAb745 and cetuximab were used, alongside the native forms. (**G** and **H**) PC9 cells were seeded in 6-well plates and subjected to overnight serum starvation. The following day, cells were treated for either 24 hours with the indicated mAbs (5, 10, and 20 µg/ml), or Bis-EM (10, 20, and 40 µg/ml), in serum-free medium or treated for 2, 4, and 8 hours with the indicated mabs (10 µg/ml) or Bis-EM (20 µg/ml). After treatment, cell lysates were collected and analyzed using an immunoblot assay that assessed receptor abundance. Vinculin was used as a gel loading control. Numbers beneath each lane show quantification of the corresponding protein bands (normalized to vinculin). (**I** and **J**) PC9 cells (3 × 10³ cells per well) were seeded in 96-well ultra-low-attachment plates and cultured overnight to form uniform spheroids. The spheroids were treated with monoclonal antibodies (mAbs; 20 μg/mL), or with the bispecific antibody Bis-EM (40 μg/mL), and monitored continuously using the IncuCyte system. Shown are epresentative spheroid images from different treatment groups at the indicated time points (I). Green fluorescence indicates caspase-3/7 activation. Culture supernatants were collected on day 5, and perforin levels in each treatment group were quantified using ELISA (J). Scale bar (in panel I), 0.8 mm. *, p<0.05. Note: Original blots corresponding to panels F, G and H can be found in Fig, S8B.

**Figure S5: Pairs of either kinase inhibitors or mAbs, both targeting either MERTK or EGFR inhibit a 3D model of osimertinib-resistant cells.** (**A**) PC9 and H1975 cells were seeded in 96-well plates. The next day, cells were treated for 72 hours using the indicated drugs. Cell viability was determined using the MTT colorimetric assay. The experiments were repeated twice using triplicates. Significance was calculated using one-way ANOVA, Dunnett’s, and Tukey’s multiple comparison tests (**, p < 0.01; ****, p < 0.0001). (**B** and **C**) An osimertinib-resistant derivative of PC9 cells (PC9-AZDR; 1x10^3^ cells per well) was seeded in BME pre-coated 96-well plates. The cells were embedded in 5% BME medium containing vehicle and or the indicated mAbs (10 μg/mL), osimertinib (10 nM), MRX-2843 (300 nM), Bis-EM (20 μg/ml), or the indicated drug combinations. The experiment was terminated after 14 days. Images of the spheroids were captured using an OpTech IB4 microscope (bright field; 20x magnification). The percentages of covered areas were assessed using ImageJ. The histograms represent the average of two independent experiments performed in duplicates. Significance was calculated using one-way ANOVA, Dunnett’s and Tukey’s multiple comparison tests (**, P < 0.01; ****, p < 0.0001). Scale bar, 50 μm.

**Figure S6: Combined inhibition of EGFR and either MERTK or AXL shows cooperative efficacy in cell line xenograft models.**

(**A** and **B**) PC9 cells (2.5 x 10^6^ per animal) were injected subcutaneously in the flanks of CD1-nu/nu mice. Once tumors became palpable, 5-9 mice were randomized and treated for five weeks with the indicated doublets (A) or triplets (B) of therapeutic agents, either mAbs (100 μg/injection) or Bis-EM (200 μg/injection, twice a week), or TKIs, osimertinib (10 mg/kg) or MRX-2843 (20 mg/kg), which were orally administered on a daily basis. Tumor volumes were monitored twice a week, and animal body weights were determined once a week. The grey-marked area denotes the treatment phase. Survival analysis was performed using Holm–Šídák’s multiple comparisons test to assess statistical significance between treatment groups. The adjusted p-value for the comparison between osimertinib alone and osimertinib combined with Bis-EM is less than 0.0013. (**C** and **D**) The indicated doublets and triplets of therapeutic agents, or combinations with Bis-EM, were tested on the H1975 xenograft model, essentially as described for panels A and B. Survival analysis was performed using Holm–Šídák’s multiple comparisons test. The adjusted p-value for the comparison between osimertinib alone and osimertinib combined with Bis-EM is less than 0.001. Shaded areas label the drug treatment phase. (**E** and **F**) H1975 cells (2.5 × 10⁶ cells per animal) were subcutaneously injected into the flanks of CD1-nu/nu mice. Once tumors became palpable, mice (n = 9 per group) were randomized and treated for five weeks with the following therapeutic agents: osimertinib (OSI), cetuximab (CTX), anti-MERTK mAb 745, anti-AXL mAb754, or the bispecufic antibodies targeting EGFR and either MERTK (Bis-EM) or AXL (Bis-EA). The monoclonal antibodies were administered at 100 μg per injection, whereas the bispecific antibodies were administered twice weekly at 200 μg per injection. Osimertinib was administered orally once daily at 10 mg/kg. Tumor volumes were measured twice weekly, and body weight was monitored once weekly throughout the treatment period. However, no overt effects of the tested drugs on animal body weight was noted. Shown are the tumor volumes we observed in individual mice (panel E) and the corresponding Kaplan–Meier survival curves (panel F). The numbers of cured mice (i.e., showing no relapses) are indicated in parentheses. For rxample, 6/9 meaning that 6 out of 9 mice were cured. For clarity and ease of comparison, the OSI+CTX+mAb745 and OSI+Bis-EM groups are reproduced from Figure 6. (**G** and **H**) PC9 cells (2.5 × 10⁶ cells/mouse) were subcutaneously injected in the right flanks of 6-week-old CD1 nude mice. When tumors became palpable, mice were randomized into four treatment groups (4 animals per group) and treated with osimertinib (10 mg/kg; daily, oral gavage) and/or the indicated antibodies (cetuximab, 0.1 mg/mouse per injection; Bis-EM, 0.2 mg/mouse per injection). Antibodies were administered twice weekly by means of intraperitoneal injection. Tumor volumes were measured throughout the treatment period. Tumor growth curves are shown in E, and the percentage change in tumor volume after 7 days of treatment is shown in F. Statistical significance was assessed using an unpaired Student t-test. *, P < 0.05, ****, P < 0.0001.

**Figure S7: When combined with the respective TKIs, the EGFRxMERTK bispecific antibody delays relapses of exon 19 and exon 20 PDX models and displays favorable safety profiles.** (**A**) The patient-derived xenograft TM00193 (E746_A750 Del19-EGFR) was engrafted subcutaneously in NSG mice. When tumors became palpable (around 350 mm^3^), mice were randomized in groups of 5-7 animals and treated for four weeks (shaded areas) with osimertinib (10 mg/kg/day; orally) and the indicated mAbs (100 micrograms/mouse/injection), or with Bis-EM (200 micrograms/mouse/injection). Tumor volumes were monitored for the next 90 days. Shown are tumor volumes per individual mice. The number of animals in each group is indicated in brackets below the name of treatment. (**B**) NSG mice were implanted with tumor fragments derived from a lung cancer PDX model, PDXJ6 (from The Jackson Laboratory, J000100672), harboring the exon 20 mutations S768_V769insSVD. Once tumors reached approximately 300 mm³, mice were randomized into groups of 5-7 animals per group. They were later treated for 3 weeks (shaded areas), twice a week, with the indicated mAbs (100 μg/injection) or with Bis-EM (200 μg/injection). Alternatively, mice were treated with the exon 20–specific TKI called poziotinib (5 mg/kg), which was administered orally once daily. Tumor volumes were monitored twice per week. Shown are the individual tumor volumes of each mouse. The number of mice per group are indicated. (**C** and **D**) PC9 cells (2.5x10^6^ per animal) were injected subcutaneously in the flanks of CD1-nu/nu mice. Once tumors became palpable, 5-9 mice were randomized and treated for four weeks with the indicated drugs. Tumor volumes and body weights were monitored. The drugs tested were either mAbs or Bis-EM (100 μg/injection or 200 μg/injection, respectively) or TKIs, either osimertinib (10 mg/kg) or MRX-2843 (20 mg/kg). Note that TKIs were orally administered once per day. Animal body weights were determined once per week. (**E** and **F**) The H1975 xenograft model was used and the results are presented essentially as described for the PC9 model in panels A and B. (**G**) NSG mice bearing subcutaneous PDX TM00193 tumors (EGFR exon-19 deletion; ~350 mm³) were randomized in groups of 5–9 animals per group. Mice were treated for 4 weeks with the indicated mAbs (100 µg/mouse; intraperitoneally, twice weekly), Bis-EM (200 µg/mouse, twice weekly), and/or osimertinib (10 mg/kg/day; orally, once per day). Body weights were monitored; 1000 mm³ was set as the sacrifice point. Changes in the animal’s body weights relative to treatment onset are shown for each treatment group. (**H**) NSG mice bearing palpable PDX6 tumors (J000100672; exon 20 mutations) were randomized in groups of 5–7 animals per group and treated for 3 weeks with the indicated antibodies, Bis-EM, or poziotinib (POZ; 5 mg/kg; orally, once daily). Body weight changes were monitored weekly throughout the experiments presented. Graphs show the average body weight for each treatment group, presented as mean volume ± SEM.

**Figure S8A:** Original blots corresponding to panels shown in Figures 5, 6 and 7.

**Figure S8B:** Original blots corresponding to panels shown in Figures S1, S2, S3 and S4..
